# Resistance versus Balance Training to Improve Postural Control in Parkinson's Disease: A Randomized Rater Blinded Controlled Study

**DOI:** 10.1371/journal.pone.0140584

**Published:** 2015-10-26

**Authors:** Christian Schlenstedt, Steffen Paschen, Annika Kruse, Jan Raethjen, Burkhard Weisser, Günther Deuschl

**Affiliations:** 1 Department of Neurology, Christian-Albrechts-University, Kiel, Germany; 2 Department of Sport Science, Christian-Albrechts-University, Kiel, Germany; University of Tuebingen, GERMANY

## Abstract

**Background:**

Reduced muscle strength is an independent risk factor for falls and related to postural instability in individuals with Parkinson’s disease. The ability of resistance training to improve postural control still remains unclear.

**Objective:**

To compare resistance training with balance training to improve postural control in people with Parkinson’s disease.

**Methods:**

40 patients with idiopathic Parkinson’s disease (Hoehn&Yahr: 2.5–3.0) were randomly assigned into resistance or balance training (2x/week for 7 weeks). Assessments were performed at baseline, 8- and 12-weeks follow-up: primary outcome: Fullerton Advanced Balance (FAB) scale; secondary outcomes: center of mass analysis during surface perturbations, Timed-up-and-go-test, Unified Parkinson’s Disease Rating Scale, Clinical Global Impression, gait analysis, maximal isometric leg strength, PDQ-39, Beck Depression Inventory. Clinical tests were videotaped and analysed by a second rater, blind to group allocation and assessment time.

**Results:**

32 participants (resistance training: n = 17, balance training: n = 15; 8 drop-outs) were analyzed at 8-weeks follow-up. No significant difference was found in the FAB scale when comparing the effects of the two training types (*p* = 0.14; effect size (Cohen’s *d)* = -0.59). Participants from the resistance training group, but not from the balance training group significantly improved on the FAB scale (resistance training: +2.4 points, Cohen’s *d* = -0.46; balance training: +0.3 points, Cohen’s *d* = -0.08). Within the resistance training group, improvements of the FAB scale were significantly correlated with improvements of rate of force development and stride time variability. No significant differences were found in the secondary outcome measures when comparing the training effects of both training types.

**Conclusions:**

The difference between resistance and balance training to improve postural control in people with Parkinson’s disease was small and not significant with this sample size. There was weak evidence that freely coordinated resistance training might be more effective than balance training. Our results indicate a relationship between the enhancement of rate of force development and the improvement of postural control.

**Trial Registration:**

ClinicalTrials.gov ID: NCT02253563

## Introduction

Postural Instability is one of the major motor symptoms of individuals with Parkinson’s disease (PD) and is generally not improved by medication or Deep Brain Stimulation [1–3]. Postural disturbances are one of the independent risk factors for falling [4–6] and fall rates range from 39 to 68% in patients suffering from PD [7,8]. Moderate evidence exists that exercise can improve postural control [9–11]. A study conducted by Canning et al. [12] showed that a combined training targeting balance, strengths and freezing of gait was effective to enhance postural control. Studies have shown that balance training (BT) alone can be effective to improve postural control in people with PD [13,14].

Resistance training (RT) is an effective treatment to improve motor symptoms [15] and strength in PD [16–19]. Reduced muscle strength coincides with an increased risk for falls in PD [4] and is associated with postural impairments [20,21]. Compensatory mechanisms play an important role in PD and the improvement of strength due to resistance training might facilitate the activation of balance related muscle-groups. Accordingly RT might lead to enhanced postural control. Two recently published meta-analysis showed no significant improvement of postural control due to RT in PD [16,17]. The authors report to interpret this result with caution as only 3 studies were analyzed having the assessment of postural control as secondary outcome [22–24]. Furthermore, none of these studies used clinical balance scales to reflect the various dimensions of postural control and the control groups did not received any intervention. Only one study had blinded rating but this study analyzed a combination of training of resistance and balance training [22]. Due to these various limitations, the effect of isolated RT on postural control still remains unclear. Interestingly, a recently published study with healthy elderly showed that RT might be efficacious, as the authors showed better improvement in postural control due to RT in comparison to classical BT [25].

In order to create the most effective exercises, studies often use multidimensional training programs [23,26,27]. These physical therapy techniques are difficult to compare and more specific trials are needed to give further information about which exercise program might be more effective and about the underlying processes leading to the results [9,28].

The aim of the present study was to compare the efficacy of RT with BT to improve postural control in people with PD. BT was chosen because classical BT is widely used in physical therapy to treat individuals with postural instability and further we wanted to pit two typical exercise interventions against each other. In addition, we intended to relate the effects on postural control with changes of several disease associated conditions in order to gain insight which mechanisms play an important role for the improvement of postural control.

## Methods

We designed a randomized rater blinded controlled trial to compare the effects of RT with the effects of BT for people with idiopathic PD. The study was registered online at ClinicalTrials.gov (ID: NCT02253563). Registration of the trial was delayed after the enrollment of the first patient due to an administrative error. The authors confirm that all ongoing and related trials for this intervention are registered.

### Participants

People were included if they met the following inclusion criteria: (1) diagnosed with idiopathic PD as defined by the UK Brain Bank criteria [29] and by a neurologist specializing in movement disorders, (2) postural instability (Fullerton Advanced Balance (FAB) scale ≤ 25 points) [30], (3) able to follow exercise instructions (assessed during a pre-examination during which the FAB scale was performed (see below)). Exclusion criteria were: (1) deep brain stimulation, (2) other diseases that could influence stance- and gait performance, (3) participation in a specific RT or BT program (beside usual physical therapy) during the last 6 months, (4) participation in any other medical, behavioral or exercise treatment (additionally to the usual received therapeutic treatment) during the study period, (5) unstable medication and (6) cardiopulmonary/metabolic diseases that could interfere with the safe conduct of the study protocol. Cognitive impairments (assessed with the Mini-Mental State Examination (MMSE)) were not defined as exclusion criteria so that a representative sample of affected patients could be included.

The study protocol was approved by the local ethics committee (Ethik-Kommission, Universitätsklinikum Schleswig-Holstein, Campus Kiel, Arnold-Heller-Straße 3, 24105 Kiel, Germany, ref. A 146/11) in September 2011 and all participants gave written informed consent prior to participating. All participants had legal capacity to make decisions and patients having a MMSE score<25 gave written informed consent together with their spouse, if applicable. The person of the images in S1 File and S2 File gave written consent to publication.

### Screening and Randomization

Participants were screened with a pre-examination prior to inclusion in the study. The FAB scale was performed to determine the level of postural instability. Patients were stratified by gender and level of postural instability and randomized in matched pairs using computer generated random number sequences in a ratio of 1:1. Participants were reassessed for baseline analysis at another day.

### Intervention

Participants were randomly assigned into RT or BT (2x/week for 7 weeks). Each session lasted 60 minutes (4–5 participants/group), and consisted of 10 minutes to warm-up followed by 50 minutes RT or BT. Each session was guided by a movement disorders experienced sport scientist who had experience in neurological rehabilitation and with the help of a sport student (student of kinesiology).

#### Resistance Training

RT was performed with the aim to improve muscle strength of the lower limbs. The trained muscle groups were hip flexors, extensors and abductors, knee flexors and extensors, ankle dorsiflexors and plantarflexors, as these are muscle groups primary involved in postural control mechanisms [20,21]. The participants’ own weight, cuff weights and elasticated bands were used as resistance [31]. Squats, knee extensions, toe/calf raises, hip abductions and other exercises were performed (see S1 File, which shows the performed exercises). In line with training recommendations based on previous studies (e.g. Hass et al. [32]) participants completed three sets of 15–20 repetitions to volitional fatigue of each exercise. With respect to the age of the participants and the stage of disease, exercise intensity was kept on a moderate level in order to avoid injuries. Once participants could complete more than 20 consecutive repetitions of an exercise, they were asked to increase the resistance to a point where they could only complete between 15–20 repetitions in order to keep the training intensity on a consistent level. Resistance was increased by cuff weights, elasticated bands or by the trainer who gave additional resistance. Participants rested for 2 minutes between exercise sets.

#### Balance Training

BT involved stance- and gait tasks which require feedforward and feedback postural control [13]. Feedforward postural control for example was trained by letting the participants lean forward, backward or sideward, thus letting them control their center of pressure inside the boundaries of their base of support. To practice feedback control one exercise was to perturb the participants by shoulder pulls from the trainer. Training progression during the intervention period was reached by reducing or manipulating sensory information, necessary to obtain balance and by adding movement to make the activity more dynamic. Visual information for example was disturbed by closing the eyes or looking up to the ceiling. Proprioceptive feedback was manipulated by standing on different unstable surfaces instead of normal overground. Each exercise lasted for 45 sec and was performed 3 times, followed by a break of 2 minutes (see S2 File, which shows the performed exercises).

### Outcome Measures

Assessments were performed at baseline, 8- and 12-weeks follow-up. Primary outcome measure was the FAB scale [33]. The FAB scale is a 10-item clinical balance scale with a 5-point ordinal scale (0–4) for each item and a maximal score of 40 points (higher values indicate better performance). The FAB scale is validated for individuals with Parkinson’s disease with excellent interrater and test-retest reliability [30]. We chose the FAB scale instead of the often used Berg Balance Scale because in contrast to the Berg Balance Scale the FAB scale has less ceiling effect and includes the assessment of reactive postural control [30]. We decided against the frequently used Mini-BESTest as the FAB scale’s items are more detailed and it takes less time to perform the FAB scale [30].

Secondary outcome measures: Center of mass (COM) displacement was analyzed during surface perturbations. Participants were asked to maintain their balance without doing steps while standing on a movable platform which shifted unexpectedly towards the anterior or posterior direction (20cm with a velocity of 0.1m/s and an acceleration of 10m/s^2^). Participants were aware neither when the platform would move nor in which direction the surface would change.COM was assessed with an infrared movement analysis system (Qualisys, Gothenburg, Sweden) consisting of six infrared cameras (240 Hz sampling rate). 17 infrared light emitting diodes were placed on anatomic landmarks as described in detail elsewhere [2] and the COM was calculated as the weighted sum of all segments, as adapted from Winter et al. [34]. According to Visser et al. [2] the vector length of three-dimensional COM displacement was calculated. In order to adapt to different biomechanical requirements due to different sizes of participants, the vector length was normalized to COM height. The average normalized vector length over all backward and forward pulls was calculated, respectively. The area under the curve of the normalized vector length from the beginning until 1 sec after the perturbation was defined as an instability outcome measure (see S3 File, which gives further details about the analysis of the surface perturbations) [2].

The following tests were used additionally: Timed-up-and-go-test (TUG) [35], Clinical Global Impression—Improvement (CGI-I) [36], Unified PD Rating Scale (UPDRS) [37], PD Questionnaire (PDQ-39) [38], Beck Depression Inventory (BDI) [39] and Physical Activity Scale for the Elderly (PASE) [40].

Gait velocity of participants was measured during uninterrupted ground level walking, recorded by light barriers placed at the beginning and at the end of a 5m pathway, which the participants had to cross 5 times. Afterwards, participants were asked to walk 2 min. on a treadmill (Woodway, Weil am Rhein, Germany) with their overground gait velocity. The treadmill comprised two separate belts, each with 4 force transducers (Kistler, Winterthur, Switzerland) (960Hz sampling rate). Contact times (heel strike, toe off) were measured by the force transducers to calculate the following spatio-temporal variables: stride length, double support time, stride time variability, bilateral coordination (Phase Coordination Index (PCI)) [41] and gait asymmetry [41] (see S4 File, which describes in detail the gait analysis).

Maximal isometric leg strength was measured on a custom designed leg press equipped with a force platform (Kistler, Winterthur, Switzerland) (1000Hz sampling rate). Maximal voluntary contraction (MVC) and rate of force development (RFD) was assessed. Results were analyzed for both legs separately according to the less- (LAS) and more (MAS) affected PD side—defined by comparing the sums of the UPDRS items 20–26 for the left and right side separately [42,43] (see S5 File, which describes in detail the strength testing).

All clinical tests were carried out by a rater who was blind to the participant’s group allocation. The FAB scale and UPDRS were videotaped and rated by a second rater, blind to participant’s group allocation and assessment time.

### Testing Procedure

Assessments were performed on two separate days. Participants were tested in the medication ON-state (1 hour after the last intake of antiparkinsonian medication). Each participant performed the baseline, 8- and 12-weeks follow-up measurements at the exact same time of day.

### Statistical Analysis

Sample size calculation was performed for the FAB scale as the main outcome. A sample size of 18 participants per group was found to be required to detect a between-group difference of 2 points at the FAB scale from baseline to 8-weeks follow-up (power = 0.9, alpha = 0.05) (G*Power, version 3.1.9 [44]). This predicted difference equate to a large effect size of 0.6 or greater. With an expected 10% drop-out rate we included 20 participants per group.

Between-group differences in demographic and baseline variables were tested using the Mann-Whitney-U-Test. Within group differences were analysed with the Wilcoxon signed rank test.

To compare the effect of treatment between the two training groups, the difference between 8-weeks follow-up and baseline performance was computed for each participant. Both groups were then compared with the Mann-Whitney-U-Test.

Non-parametric statistical tests were used for the demographic, within and between group analyses as some of the outcome variables are ordinal scaled and not all of the variables were normally distributed.

The Mann-Whitney-U-Test was used to compare CGI-I between the groups.

Interrater reliability between the blinded rater and the blinded video rater were analysed by calculating two-way mixed single measure intraclass correlation coefficients (ICC (3,1)).

Cohen’s *d* was calculated to evaluate effect sizes.

To analyze the relationship between the magnitude of change in the different outcome variables, Spearman’s rank correlation coefficients (Spearman’s Rho) were calculated. Those variables which significantly correlated with the changes in the FAB scale were included as independent variables in a multiple linear regression analysis. To analyze the risk of multicollinearity variance inflation factors were calculated for each independent variable. A variance inflation factor > 10 indicates high multicollinearity [45].

Data were analyzed on a per-protocol basis. Participants were excluded if they missed more than two training sessions, if medication was changed or if any other injury which could influence stance- and gait performance occurred during the study period.

Statistical tests were performed with SPSS (version 19, IBM), the α level for significance was set at *P* < 0.05 and all tests were two-sided. Bonferroni correction was used for multiple comparisons for the variables of the gait analysis and strength testing separately.

The study protocol and supporting CONSORT checklist are available as supporting information (S1 Protocol, S2 Protocol, and S1 CONSORT Checklist).

## Results

**Fig 1:**
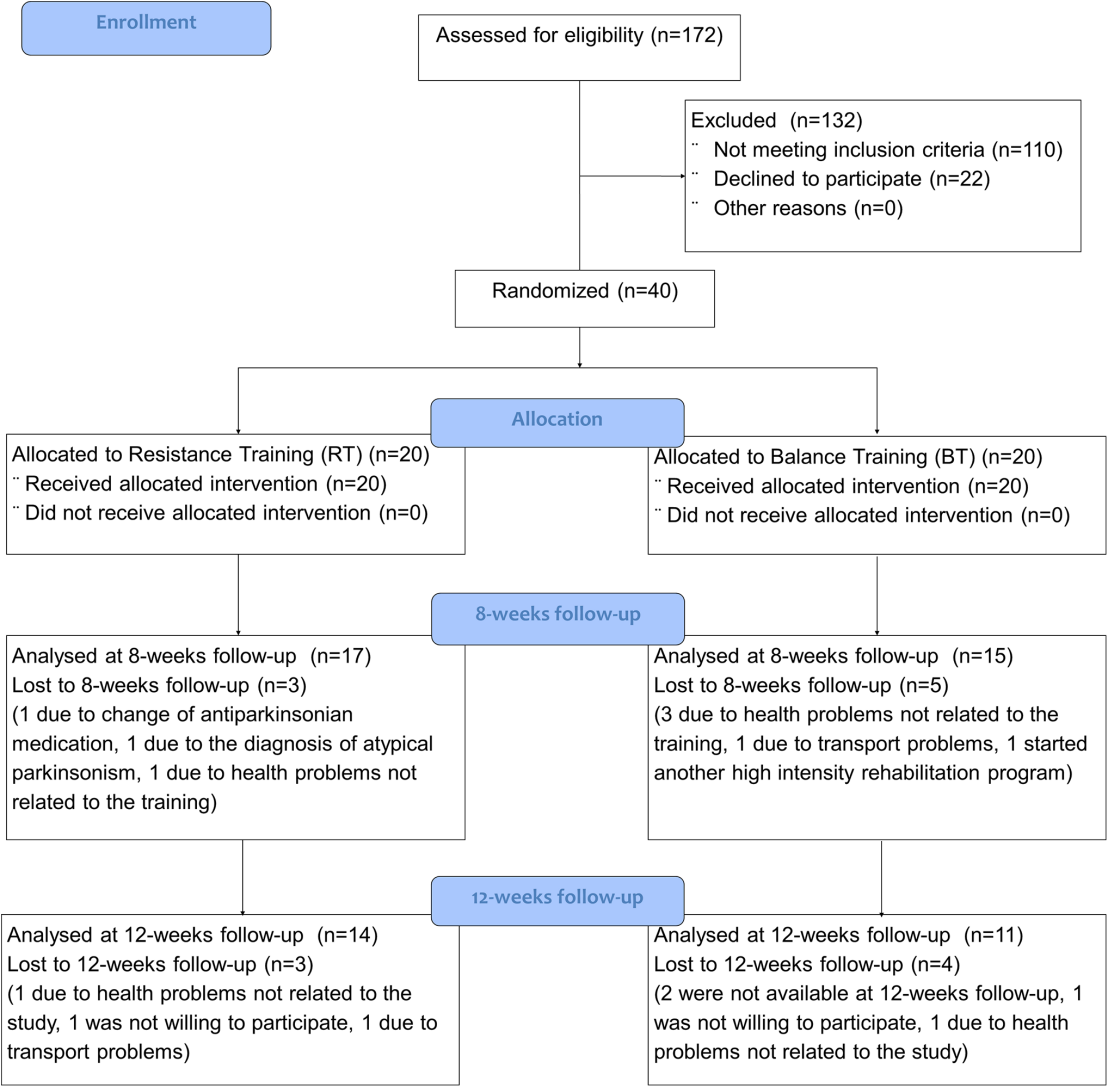
The CONSORT flow diagram for this study.

From September 2011 till August 2013 a total of 172 persons were screened for eligibility at the department of Neurology, University Hospital Schleswig-Holstein, Kiel, Germany, among which 40 patients met the inclusion criteria and underwent randomization. Final data collection was February 2014. 8 participants (20%; 3 RT; 5 BT) did not complete the training protocol. For drop-out reasons see Fig 1 which shows the CONSORT flow diagram. All patients were able to follow the instructions during the training sessions.

### Baseline data

No significant differences were found in the demographic or baseline variables between the two groups except for the outcome forward pull (Tables 1 and 2).

**Table 1:** Participant characteristics.

Variable	Resistance (n = 17)	Balance (n = 15)	*p-*value^a^
Age (yr)	75.7 ± 5.5	75.7 ± 7.2	0.882
No. of female subjects	5 (29.4%)	6 (40%)	0.529^b^
Duration of Disease (yr)	10.1 ± 6.0	9.3 ± 7.9	0.455
BMI (kg/m^2^)	27.9 ± 5.5	25.5 ± 4.4	0.142
H&Y	2.8 ± 0.26	2.7 ± 0.4	0.216
H&Y (Range)	2.5–3.0	2.5–3.0	n/a
UPDRS total (ON)	40.2 ± 12.5	37.7 ± 13.1	0.455
UPDRS part II (ON)	13.4 ± 5.1	11.1 ± 6.0	0.105
UPDRS part III (ON)	23.6 ± 9.5	22.3 ± 6.1	0.941
FAB scale	22.2 ± 4.8	24.5 ± 4.6	0.123
MMSE	27.3 ± 3.6	27.7 ± 3.0	0.891
MMSE (Range)	17–30	20–30	n/a
PASE score	104.6 ± 87.3	77.2 ± 63.1	0.576
LEDD (mg/day)	817.4 ± 468.0	674.7 ± 294.9	0.318

If not indicated differently, values are either mean ± SD or number and percentage. BMI, Body-Mass-Index; FAB, Fullerton Advanced Balance; H&Y, Hoehn & Yahr; LEDD, levodopa equivalent daily dose; MMSE, Mini-Mental State Examination; PASE, Physical Activity Scale for the Elderly; UPDRS, Unified Parkinson’s Disease Rating Scale

^a^ unless otherwise indicated *P*-value of independent samples Mann-Whitney-U-Test

^b^ *P*-value of Chi-Square Test.

**Table 2:** Comparison within- and between the two training groups from baseline to 8-weeks follow-up.

Variable		Baseline	8-wk follow-up	Mean change (95% CI) from baseline to 8-wk follow-up	*p-*value^a^ (within group comparison)	*p-*value^b^ (between group comparison)	Effect size^c^ (within group)	Effect size^c^ (between group)
FAB scale^d^	RT	22.2 ± 4.8	24.5 ± 5.4	2.4 (0.1; 4.6)	0.04*	0.143	-0.46	-0.59
	BT	24.5 ± 4.6	24.9 ± 5.3	0.3 (-0.8; 1.5)	0.526		-0.08	
Forward pull^e^	RT	2270.3 ± 375.1	2336.7 ± 274.0	66.4 (-138.2; 270.9)	0.311	0.769	-0.20	-0.17
	BT	1807.4 ± 351.8	1836.6 ± 360.9	29.2 (-66.4; 124.9)	0.239		-0.08	
Backward pull^e^	RT	1882.3 ± 326.9	1782.1 ± 373.4	-100.2 (-311.5; 111.0)	0.286	0.332	0.29	0.42
	BT	1844.6 ± 411.4	1917.4 ± 362.7	72.8 (-261.4; 407.0)	0.657		-0.19	
TUG (sec)	RT	11.2 ± 3.2	9.5 ± 2.4	-1.7 (-3.3; -0.1)	0.033*	0.139	0.60	0.69
	BT	9.2 ± 3.0	9.0 ± 1.8	-0.2 (-1.3; 0.9)	0.929		0.08	
UPDRS total score	RT	40.2 ± 12.5	38.5 ± 12.3	-1.7 (-5.1; 1.8)	0.347	0.272	0.14	-0.38
	BT	37.7 ± 13.1	33.6 ± 12.3	-4.1 (-7.3; -0.9)	0.033*		0.32	
UPDRS motor score^f^	RT	22.6 ± 8.8	22.2 ± 8.9	-0.4 (-2.0; 1.2)	0.568	0.911	0.04	-0.51
	BT	20.3 ± 4.9	19.4 ± 6.7	-0.9 (-3.0; 1.1)	0.821		0.49	
gait velocity (cm/sec)	RT	104.3 ± 15.3	106.1 ± 15.0	1.8 (-5.2; 8.7)	0.619	0.692	-0.12	-0.14
	BT	106.9 ± 18.3	106.8 ± 17.7	-0.1 (-7.4; 7.4)	0.776		0.01	
stride length (cm)	RT	80.6 ± 13.0	80.3 ± 11.7	-0.4 (-4.5; 3.7)	0.865	0.097	0.02	0.50
	BT	88.8 ± 15.7	91.5 ± 16.1	2.7 (-0.4; 5.9)	0.131		-0.17	
double support time (msec)	RT	156.6 ± 31.7	156.3 ± 35.5	-0.3 (-8.4; 7.8)	0.532	0.134	0.01	0.45
	BT	149.4 ± 24.9	155.0 ± 32.1	5.6 (-2.1; 13.3)	0.11		-0.19	
stride time variability (%)	RT	3.8 ± 1.0	3.7 ± 1.7	-0.1 (-0.8; 0.6)	0.334	0.413	0.07	-0.53
	BT	3.9 ± 1.8	3.0 ± 0.8	-0.9 (-2.0; 0.3)	0.182		0.65	
PCI (%)	RT	6.6 ± 1.5	6.1 ± 1.8	-0.5 (-1.1; 0.1)	0.061	0.077	0.30	0.75
	BT	6.1 ± 1.4	6.9 ± 2.1	0.8 (-0.7; 2.3)	0.286		-0.45	
Asymmetry Index	RT	5.1 ± 4.1	6.0 ± 4.3	0.9 (-1.1; 0.1)	0.82	0.959	-0.21	-0.22
	BT	4.9 ± 3.7	5.0 ± 5.3	0.1 (-1.6; 1.8)	0.99		-0.02	
leg strength (MVC), LES (N)	RT	393.8 ± 113.5	416.9 ± 91.0	23.0 (-15.5; 61.6)	0.279	0.458	-0.22	-0.43
	BT	416.5 ± 129.6	408.8 ± 138.5	-7.7 (-53.3; 37.8)	0.925		0.06	
leg strength (MVC), MAS (N)	RT	401.8 ± 130.0	399.8 ± 85.7	-2.0 (-48.2; 44.2)	0.807	0.287	0.02	0.33
	BT	407.9 ± 134.4	426.2 ± 131.6	18.3 (-4.4; 41.1)	0.133		-0.14	
peak RFD, LAS (N/msec)	RT	1.5 ± 0.7	1.6 ± 0.8	0.1 (-0.4; 0.4)	0.753	0.223	-0.13	0.50
	BT	1.6 ± 1.0	1.8 ± 0.9	0.3 (0.0; 0.5)	0.028**		-0.21	
peak RFD, MAS (N/msec)	RT	1.5 ± 1.0	1.5 ± 0.7	0.0 (-0.6; 0.5)	0.972	0.503	0.00	0.40
	BT	1.4 ± 0.7	1.7 ± 0.9	0.3 (-0.2; 0.8)	0.308		-0.37	
RFD, LAS (N/msec)	RT	0.8 ± 0.6	0.9 ± 0.6	0.1 (-0.2; 0.5)	0.249	0.627	-0.17	0.31
	BT	0.7 ± 0.5	1.0 ± 0.6	0.3 (0.0; 0.5)	0.056		-0.54	
RFD, MAS (N/msec)	RT	0.8 ± 0.7	0.9 ± 0.6	0.1 (-0.3; 0.5)	0.600	0.939	-0.15	-0.14
	BT	0.9 ± 0.7	0.9 ± 0.7	0.0 (-0.4; 0.4)	0.507		0.00	

FAB, Fullerton Advanced Balance; TUG, Timed-Up-and-Go-Test; LAS, less affected side; MAS, more affected side; MVC, maximal voluntary contraction; PCI, Phase Coordination Index; RFD, rate of force development (0-100ms); UPDRS, Unified Parkinson’s Disease Rating Scale

^a^ *p*-value of Wilcoxon test

^b^ *p*-value of independent samples Mann-Whitney-U-Test

^c^ Cohen’s d was calculated as effect size

^d^ blinded video rating

^e^ values represent the area under the curve of the normalized vector length from 0–3 sec after the surface perturbation

^f^ blinded video rating, without item 22 (rigidity)

RT, resistance training (n = 17); BT, balance training (n = 15)

*significant different (p<0.05)

**after Bonferroni-adjustment not significant.

### Agreement between the two blinded raters

The agreement between the blinded rater and the blinded video rater was high with ICCs >0.80 for baseline and 8-weeks follow-up. Since the blinded video rater (the person who rated by videos) not only was blind to group allocation but also to assessment time, results are analysed and interpreted with priority to the blinded video rater.

### Effect of intervention from baseline to 8-weeks follow-up

The RT-group significantly improved from baseline to week 8 on average by 2.4 points on the FAB scale (*p* = 0.04; Cohen’s *d* = -0.46), whereas the score of the BT-group only increased on average by 0.3 points and that was statistically not significant (*p* = 0.526; Cohen’s *d* = -0.08) (Table 2). The higher intervention effect of the RT-group did not differ significantly from the training effect of the BT-group (*p* = 0.143, Cohen’s *d* = -0.59).

**Fig 2:**
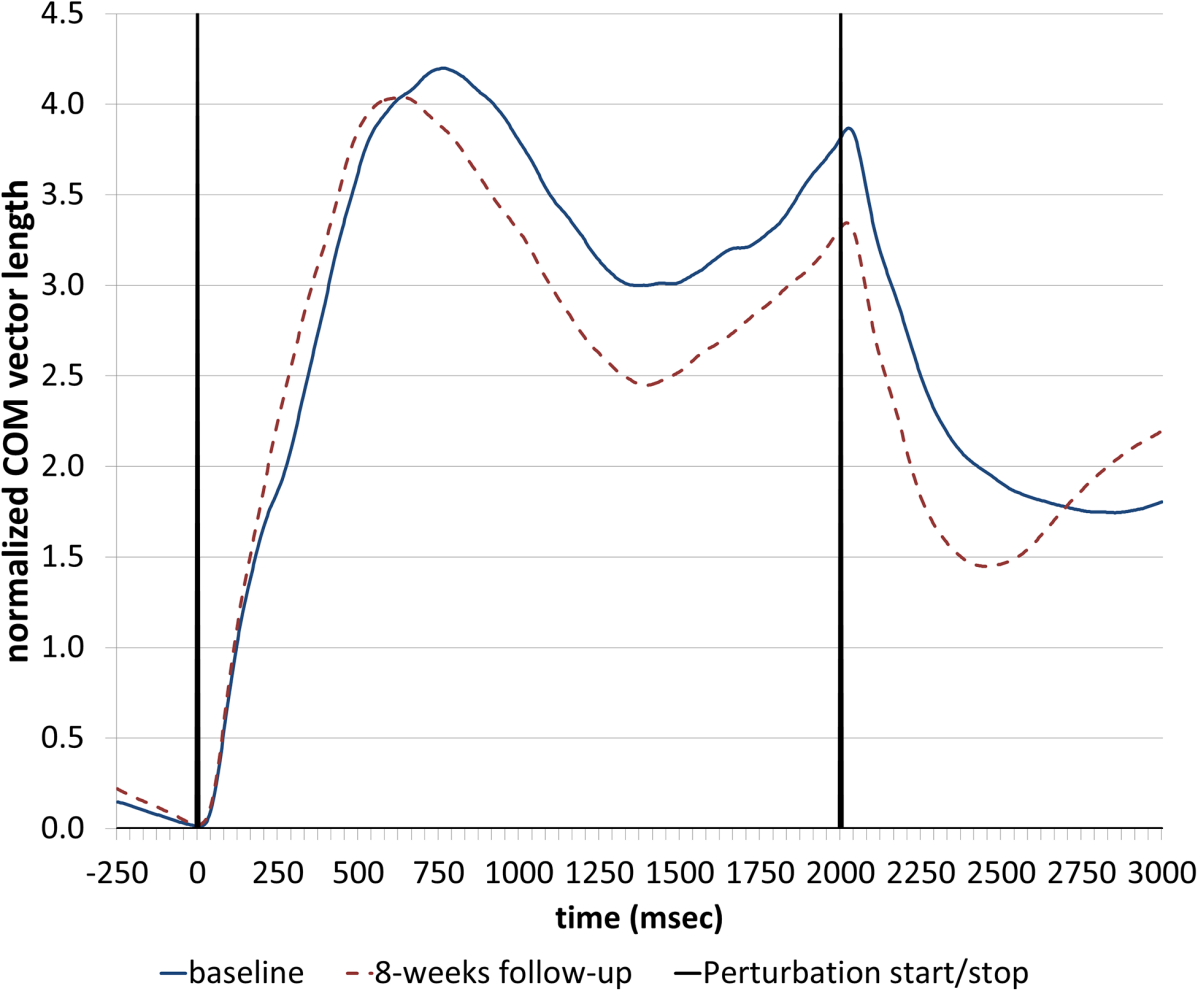
Average normalized center of mass (COM) vector length of one participant during backward perturbations at baseline and 8-weeks follow-up.

No significant differences were found when analysing the COM displacement during surface perturbations (an example of the average normalized COM vector length during backward perturbations is shown in Fig 2).

The RT-group but not the BT-group performed the TUG significantly quicker at 8-weeks follow-up in comparison to baseline (on average -1.7sec, *p* = 0.033) but the difference between the training types was not significant (*p* = 0.139).

**Fig 3:**
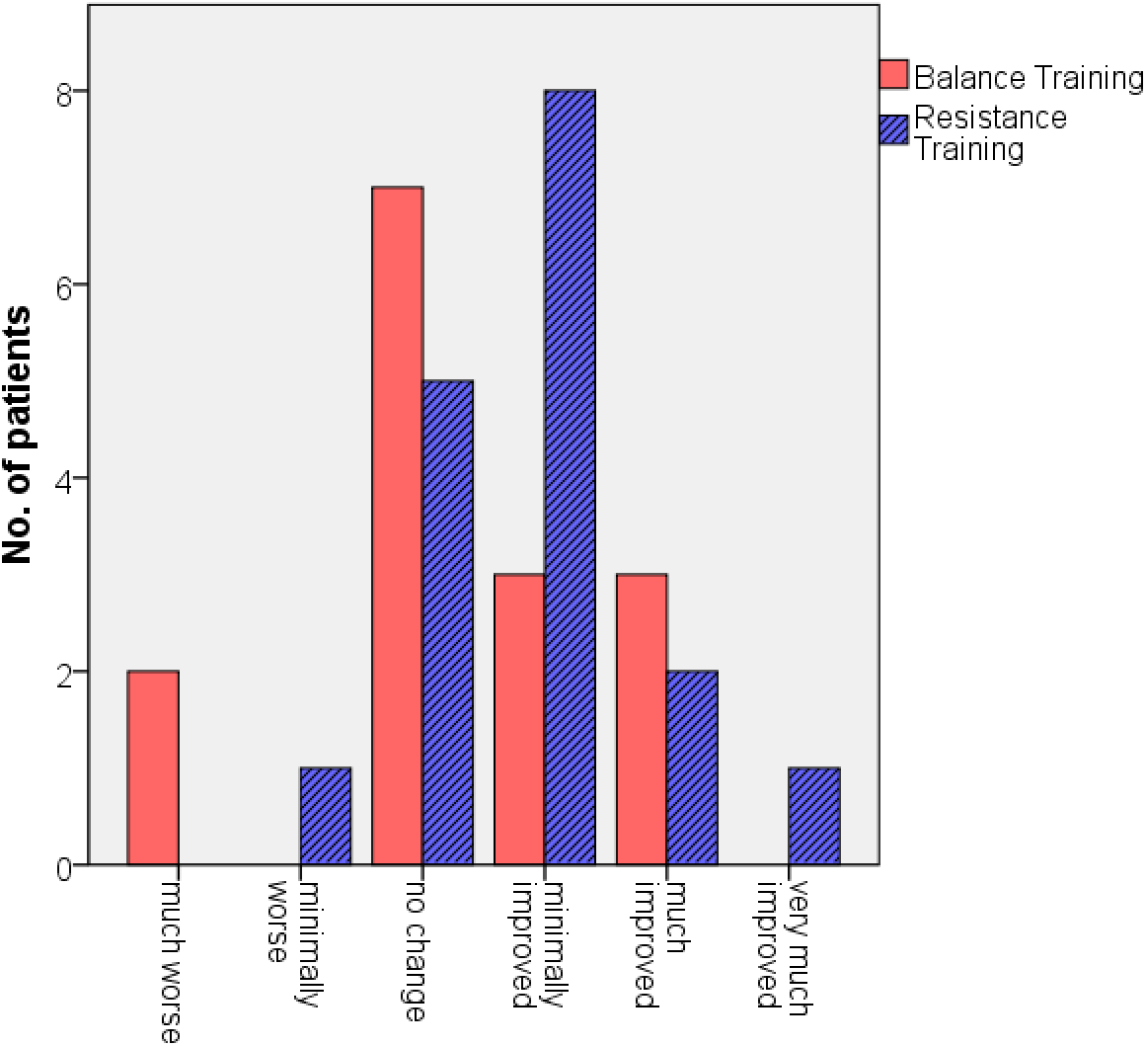
Results of the Clinical Global Impression–Improvement scale (CGI-I).

Fig 3 shows the results of the CGI-I. 65% of the participants from the RT-group reported a clinical global improvement whereas only 40% of the participants from the BT-group indicated amelioration. However, the difference between both groups was not significant (*p* = 0.295).

In contrast to the RT-group, a significant improvement was found for the BT-group at the UPDRS total score (on average -4.1 points, *p* = 0.033) without any significant difference between the training types (*p* = 0.272). No significant differences were found for the UPDRS motor score.

The BT-group slightly improved the peak rate of force development of the less affected side from baseline to week 8, but this improvement was statistically not significant after Bonferroni-correction.

No significant differences were found for the PDQ-39 (baseline: RT: 28.5 ±12.7, BT: 28.5 ±17.7; 8-weeks follow-up: RT: 26.5 ±12.0, BT: 30.2 ±17.8) and the BDI (baseline: RT: 9.9 ±5.6, BT: 14.0 ±9.1; 8-weeks follow-up: RT: 8.4 ±5.3, BT: 10.8 ±5.9) for the between group comparison (baseline and differences from baseline to 8-weeks follow-up) and within group comparison (*p*<0.05).

### Effect of intervention from baseline to 12-weeks follow-up

Table 3 shows the results of the baseline, 8- and 12 weeks follow-up assessments. The differences within one group from baseline to week 12 and the comparison of changes between the two training groups were statistically not significant.

**Table 3:** Comparison of baseline, 8- and 12-weeks follow-up.

Variable	Group	Baseline	8-wk follow-up	12-wk follow-up	Mean change (baseline to 12-wk follow-up) (95% CI)	*p*-value^a^ (within group comparison, baseline to 12-wk follow-up)	*p*-value^b^ (between group comparison, baseline to 12-wk follow-up)
FAB scale^c^	RT	22.2 ± 5.3	24.4 ± 5.7	22.5 ± 5.1	0.3 (-2.2; 2.8)	0.370	0.767
	BT	24.8 ± 4.2	25.3 ± 4.4	24.0 ± 4.6	-0.8 (-2.6; 1.0)	0.900	
TUG (sec)	RT	11.4 ± 3.6	9.4 ± 2.5	10.0 ± 2.1	-1.4 (-3.2; 0.4)	0.686	0.699
	BT	9.2 ± 3.8	9.0 ± 2.4	8.4 ± 1.9	-0.8 (-3.5; 1.9)	0.139	
UPDRS total score	RT	40.7 ± 15.0	40.7 ± 13.1	39.4 ± 12.0	-1.4 (-9.8; 7.1)	0.183	0.797
	BT	38.8 ± 14.7	32.8 ± 13.5	36.4 ± 15.9	-2.5 (-9.0; 4.1)	0.286	
UPDRS motor score^d^	RT	23.7 ± 10.4	23.4 ± 10.5	22.5 ± 10.2	-1.2 (-3.3; 0.9)	0.183	0.833
	BT	20.8 ± 4.1	19.3 ± 6.6	19.6 ± 5.5	-1.3 (-3.4; 0.9)	0.052	

FAB, Fullerton Advanced Balance; TUG, Timed-Up-and-Go-Test; UPDRS, Unified Parkinson’s Disease Rating Scale

^a^ *p*-value of Wilcoxon test

^b^ *p*-value of independent samples Mann-Whitney-U-Test

^c^ blinded video rating

^d^ blinded video rating, without item 22 (rigidity)

RT, resistance training (n = 14); BT, balance training (n = 11).

### Correlation between different outcome variables

When correlating the differences from baseline to week 8 of the FAB scale (∆-FAB scale) with the magnitude of changes of the other test variables, we found significant correlations between ∆-FAB scale and ∆-stride time variability (Spearman’s Rho: -0.649, *p* = 0.009) and ∆-RFD (LES) (Spearman’s Rho: 0.643, *p* = 0.018) within the RT-group (Table 4). A multiple linear regression analysis showed that 71.6% (adjusted R^2^) of the variance of ∆-FAB scale (as dependent variable) can be explained by ∆-stride time variability and ∆-RFD (LES) (as independent variables) and this model was statistically significant (F = 14.9, *p* = 0.001). Stride time variability and average RFD equally contributed to the model (stride time variability: Beta = 0.517, T = 2.98, *p* = 0.015; RFD: Beta = -0.54, T = -3.114, *p* = 0.012).

**Table 4:** Correlation between the differences from baseline to 8-weeks follow-up of the FAB scale with the differences from baseline to 8-weeks follow-up of other outcomes.

	Resistance		Balance	
Variable	Rho^a^	*p*	Rho^a^	*p*
Forward pull^b^	-0.318	0.289	-0.366	0.241
Backward pull^b^	-0.217	0.420	0.019	0.956
TUG	-0.097	0.754	0.230	0.497
UPDRS total score	-0.413	0.100	0.003	0.992
UPDRS motor score^c^	-0.397	0.115	0.030	0.915
gait velocity	0.148	0.572	0.058	0.837
stride length	0.319	0.246	-0.074	0.828
double support time	0.310	0.260	-0.357	0.281
stride time variability	**-0.649**	**0.009**	0.260	0.440
PCI	-0.152	0.587	**-0.608**	**0.047**
Asymmetry Index	0.215	0.441	-0.153	0.653
Leg Strength (MVC), LAS	0.014	0.964	0.343	0.230
Leg Strength (MVC), MAS	-0.510	0.075	-0.140	0.647
peak RFD, LAS	0.114	0.712	-0.003	0.993
peak RFD, MAS	0.263	0.385	-0.119	0.713
average RFD (0-100ms), LAS	**0.643**	**0.018**	0.276	0.340
average RFD (0-100ms), MAS	0.355	0.235	-0.174	0.569
PDQ-39	0.017	0.948	0.053	0.852
BDI	0.337	0.186	**0.718**	**0.003**

BDI, Beck Depression Inventory; FAB, Fullerton Advanced Balance; TUG, Timed-Up-and-Go-Test; LAS, less affected side; MAS, more affected side; MVC, maximal voluntary contraction; PCI, Phase Coordination Index; PDQ-39, Parkinson’s Disease Questionnaire; RFD, rate of force development; UPDRS, Unified Parkinson’s Disease Rating Scale

^a^ Spearman’s rank correlation coefficient

^b^ value represents the area under the curve of the normalized vector length from 0–3 sec after the surface perturbation (see Fig 2 and S3 File)

^c^ blinded video rating, without item 22 (rigidity).

Within the BT-group significant correlations were found between ∆-FAB-scale and ∆-PCI (Spearman’s Rho: -0.608, *p* = 0.047) and ∆-BDI (Spearman’s Rho: 0.718, *p* = 0.003) (Table 4). With ∆-PCI and ∆-BDI as predictors for ∆-FAB-scale in the multiple linear regression analysis for the BT-group, the model failed to be significant (adjusted R^2^ = 19.2%; F = 2.191, *p* = 0.174).

The independent variables of both models did not correlate (Spearman’s Rho<0.6; p<0.05) and the variance inflation factors were below 2.2 indicating a very low risk of mutlicollinearity.

No significant correlation was found when correlating the degree of cognitive impairment (measured by the MMSE) and ∆-FAB scale.

## Discussion

No significant differences were found when comparing the effects of RT with the effects of BT to improve postural control in individuals with PD. Within the RT group, participants significantly improved postural control with a medium effect size. The average improvement at the FAB scale of the RT group was beyond the minimal detectable change (MDC) (MDC_95_ = 2.25 points, calculated according to [30,46]), indicating a true performance change instead of a change due to variability of performance or measurement error. Participants from the BT group only slightly improved on the FAB scale but this amelioration was not significant and the effect size was small. Within the RT group 7 patients improved beyond the MDC of the FAB scale whereas only 2 of the participants of the BT group showed improvements beyond the MDC. The fact, that the difference between the training effects was not significant, may be due to our small sample size. We conclude that there exists only a small difference between RT and BT. With regard to the large effect size when comparing the effects of the two training interventions, a tendency is given that RT might be more effective than BT to improve postural control in this population.

It has been shown that balance training can be effective to improve postural control in PD [13,14]. These studies used higher training frequencies which may explain the different findings in comparison to our trial. Furthermore the aim of these studies was not to compare competing training types but to analyse the efficacy of one training type.

The higher training effects of the RT group in comparison to the BT group on the FAB scale is notable, as—in contrast to RT—the items of the FAB scale are closely related to the exercises of the BT. All participants underwent an examination to assess eligibility before participating meanwhile the FAB scale was carried out the first time. At baseline the participants thus performed the scale the second time. This emphasizes to consider the improvement from baseline to week 8 due to training effects and not based on memory effects due to the repetition of the same test.

It has to be taken into account that participants only trained two times per week. Training frequency therefore was low and maybe not high enough to detect significant differences. The pre-intervention level of physical activity of the participants was relatively low but similar to the activity level of healthy age-matched controls. In the study of Joshua et al. [25] who showed significant stronger improvement in postural control due to RT in comparison to BT in healthy elderly, training intensity was much higher and participants trained 4x/week for 6 months. As our participants were in an advanced stage of disease (H&Y: 2.5–3.0) and all of them reported to have postural impairments, we considered this training frequency practical feasible as most of the participants were not able to come to the training sessions alone and probably may not be able to train more often.

Gait velocity did not improve due to RT. This is in line with a recently published meta-analysis [16]. Furthermore, we have shown that stride lengths, double support time, gait variability, gait asymmetry and bilateral coordination did not improve due to both training types. To our best knowledge, this is the first study analysing the efficacy of RT and BT on more specific gait features than gait velocity.

The relationship between the improvement in postural control and improvement in rate of force development of the less affected PD site highlights the importance of strength with regard to postural control. The ability to generate force in the early onset of muscle contraction seems to play an important role for postural control mechanisms. By contrast, the changes of overall motor and mobility performance (measured by the UPDRS and TUG) did not correlate with the improvements of balance. The fact that especially the RFD of the less- but not the more affected PD side contributed to better postural control is in accordance with a recent study showing that training the less affected side leads to higher improvements in PD than standard exercise [47]. This raises the idea that RT may be an effective compensatory strategy to enhance postural control in this population.

RT was not performed with exercise machines; instead, participants’ body weight, cuff weights and elasticated bands were used as resistance. This was done as we wanted to perform a training type, which is–as BT–easy and cost-effective to perform without the need of exercise machines which are not always present in physical therapy. We are aware that beside the main aim to improve strength, RT with freely coordinated exercises may train sensorimotor integration as well. However, the primary objective of these kinds of exercises is the improvement of muscle strength.

Three participants had MMSE scores below 25 points. As some tests with multi-step instructions (FAB scale and TUG) and some tests with self-report measures (UPDRS, BDI, PDQ-39 and CGI-I) require cognitive capacity, we reanalyzed our data excluding these three patients for the between group comparison. Results did not change except for the PDQ-39 (significant higher improvement of the RT group in comparison to the BT group).

The following limitations exist. First, one major limitation is that training frequency was low and probably under-dosed to detect significant differences between these two competing training types. Second, we had a 20% drop-out rate, which was larger as we anticipated in the sample size calculation. Our sample size therefore might have been underpowered to detect significant differences. Especially as the correlation- and regression analysis were performed with the RT- and BT-group separately, results have to be interpreted with caution with respect to the small sample size. Furthermore, we did not assess fall rates which would be of interest as strength and balance performance are independent risk factors for falls. Finally, we did not include any control group without any intervention which would allow to further interpret the effects of both training types.

## Conclusions

This randomized controlled rater blinded trial shows that the difference between RT and BT to improve postural control in individuals with PD was small and not significant with this sample size. There was weak evidence that freely coordinated RT might be more effective than BT. Our results indicate a relationship between the enhancement of rate of force development and the improvement of postural control within the RT group, but this should be verified in future trials. Future studies should include larger sample sizes to further explore the impact of RT to improve postural control in patients with PD. The comparison of competing training interventions should be analyzed furthermore to gain insight into which exercise program might be most effective and about the underlying processes leading to the results. Concerning long-term attendance the assessment of how much the participants like to participate in a specific training type should be included.

## Supporting Information

S1 CONSORT Checklist CONSORT checklist. (PDF)

S1 File Resistance training. Text and figures which describe the resistance training.
(PDF)

S2 File Balance training. Text and figures which describe the balance training.
(PDF)

S3 File Surface perturbations. Text which gives further details about the surface perturbations.
(PDF)

S4 File Gait analysis. Text which gives further details about the gait analysis.
(PDF)

S5 File Strength testing. Text which gives further details about the strength testing.
(PDF)

S1 Protocol Study Protocol (English translation). (PDF)

S2 Protocol Study Protocol. (PDF)

## Abbreviations:

BDI: Beck Depression Inventory
BT: Balance Training
CGI-I: Clinical Global Impression–Improvement
FAB: Fullerton Advanced Balance
ICC: intraclass correlation coefficient
LAS: less affected Parkinson’s disease side
LSWT: long swing time
MAS: more affected Parkinson’s disease side
MDC: minimal detectable change
MMSE: Mini-Mental State Examinaion
MVC: maximal voluntary contraction
PD: Parkinson’s disease
PDQ-39: Parkinson’s disease Questionnaire
PASE: Physical Activity for the Elderly
RFD: rate of force development
RT: Resistance Training
SSWT: short swing time
TUG: Timed-up-and-go-test
UPDRS: Unified Parkinson’s Disease Rating Scale
