# Supplementary material for: Resistance versus Balance Training to Improve Postural Control in Parkinson's Disease: A Randomized Rater Blinded Controlled Study
Source: PLoS One. 2015 Oct 26;10(10):e0140584. doi: 10.1371/journal.pone.0140584 (PMC4621054; doi:10.1371/journal.pone.0140584)
Supplement: S1 File — Text and figures which describe the resistance training. (PDF) [file pone.0140584.s002.pdf]

Resistance- versus Balance Training to improve postural control in Parkinson's Disease

Christian Schlenstedt[1,2], Steffen Paschen[1], Annika Kruse[2], Jan Raethjen[1], Burkhard Weisser[2], Günther Deuschl[1]

[1] Department of Neurology, Christian-Albrechts-University, Kiel, Germany
[2] Department of Sport Science, Christian-Albrechts-University, Kiel, Germany

Supporting Information 1

Resistance Training

Subjects received 14 sessions (7 weeks, twice a week) of group exercise training with 4-5 participants per group. Each session lasted 60 minutes, 10 minutes warm-up followed by 50 minutes resistance training. In each session 4 of the following exercises were performed. Participants had to complete three sets of 15 – 20 repetitions of each exercise. Once participants could complete more than 20 consecutive repetitions of an exercise, participants were asked to increase the resistance to a point where they could only complete between 15 – 20 repetitions. Resistance was increased by cuff weights, elasticated bands or by the therapist who gave additional resistance. Participants rested for 2 minutes between exercise sets.

1. Squats

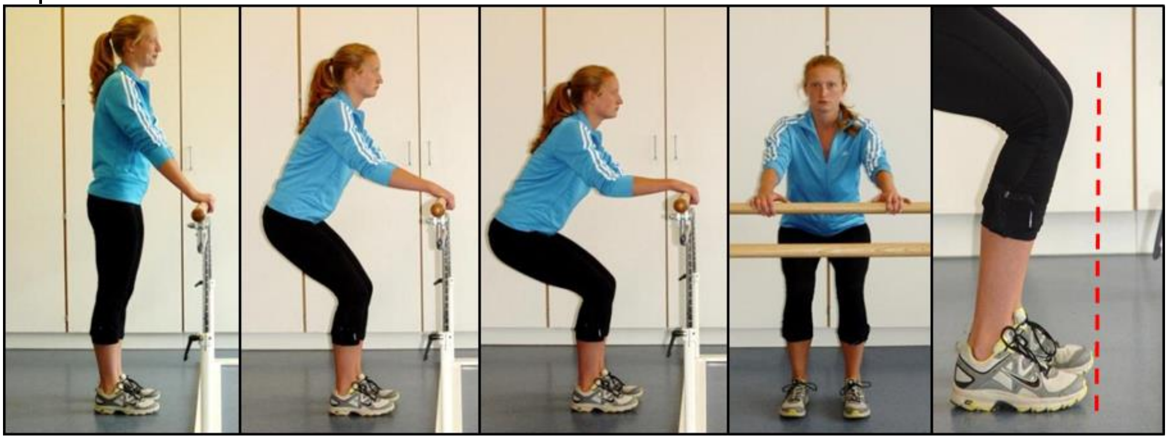

2. Knee extension (with cuff weight)

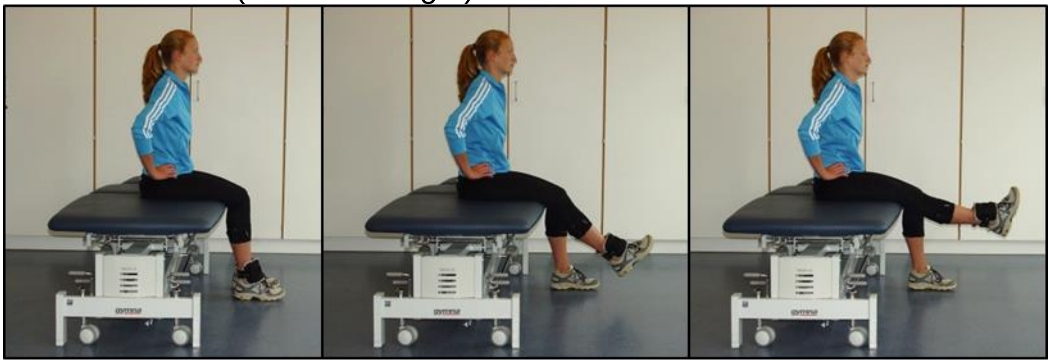

3. Calf raises

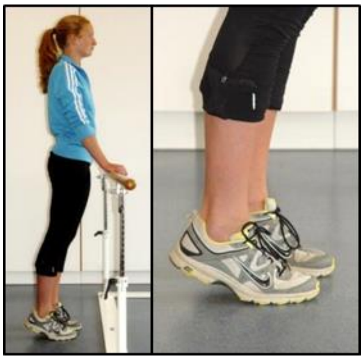

4. Toe raises (and hip flexion) (with elasticated band)

a)

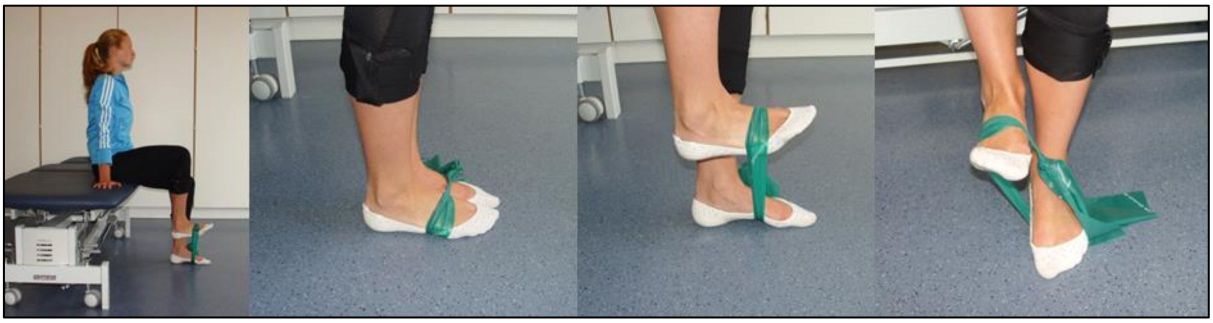

b)

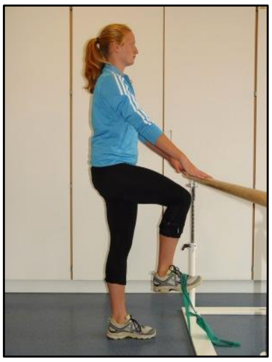

5. Hip flexion (with cuff weight)

a)

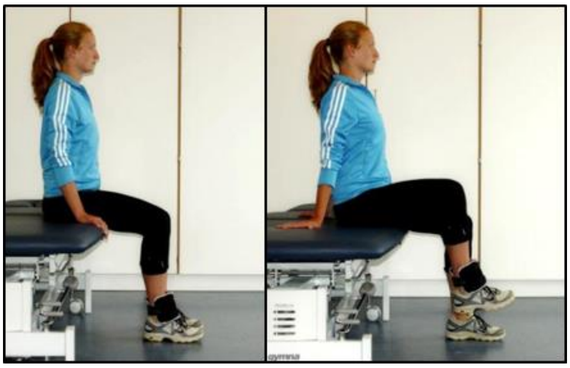

b)

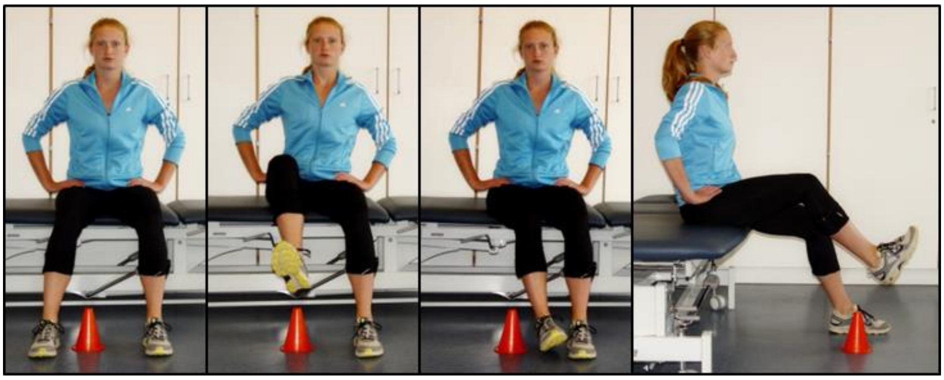

6. Hip abduction

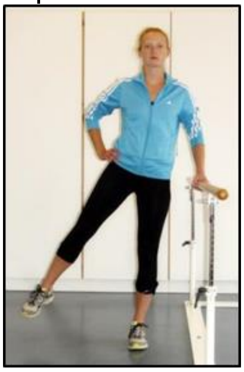

7. Ischiocrural muscles (with elasticated bands)

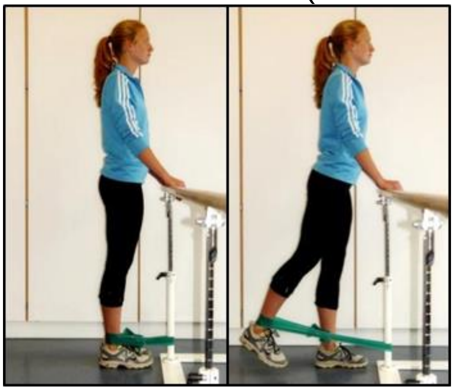

The person of the images gave written consent to publication.
