# Supplementary material for: Resistance versus Balance Training to Improve Postural Control in Parkinson's Disease: A Randomized Rater Blinded Controlled Study
Source: PLoS One. 2015 Oct 26;10(10):e0140584. doi: 10.1371/journal.pone.0140584 (PMC4621054; doi:10.1371/journal.pone.0140584)
Supplement: S2 File — Text and figures which describe the balance training. (PDF) [file pone.0140584.s003.pdf]

Resistance- versus Balance Training to improve postural control in Parkinson's Disease

Christian Schlenstedt[1,2], Steffen Paschen[1], Annika Kruse[2], Jan Raethjen[1], Burkhard Weisser[2], Günther Deuschl[1]

[1] Department of Neurology, Christian-Albrechts-University, Kiel, Germany
[2] Department of Sport Science, Christian-Albrechts-University, Kiel, Germany

Supporting Information 2

Balance Training

Subjects received 14 sessions (7 weeks, twice a week) of group exercise training with 4-5 participants per group. Each session lasted 60 minutes, 10 minutes warm-up followed by 50 minutes balance training. Each exercise was hold for 45 sec and performed 3 times, followed by a break of 2 minutes. Training progression during the intervention period was reached by reducing or manipulating sensory information, necessary to obtain balance. Visual information for example was disturbed by closing the eyes or looking up to the ceiling. Proprioceptive feedback was manipulated by standing on different unstable surfaces instead of normal overground. Subjects trained on normal overground during week 1-3 and on unstable surfaces during week 4-7.

A) Stance on normal overground

1. Tandem stance

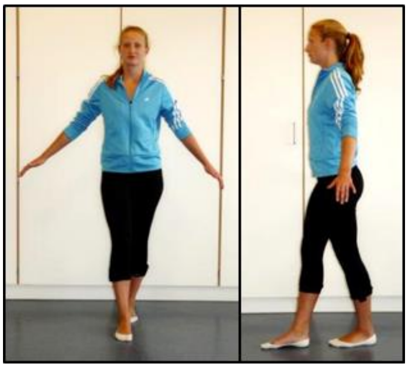

2. One leg stance:
   a) normal
   b) one foot clamped to the supporting leg
   c) the free leg has to move a rope in a circle
   d) the free leg has to move a ball in different directions

a) 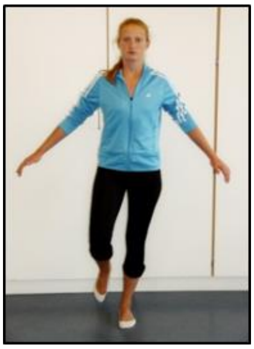 b) 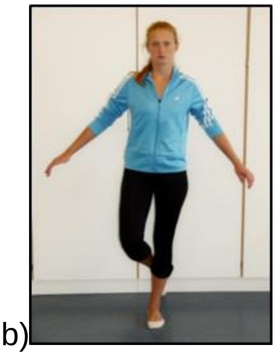 c) 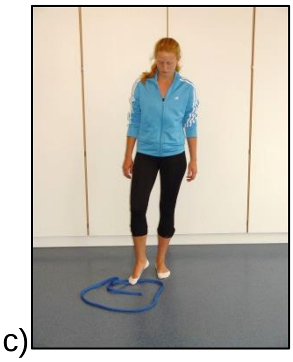 d) 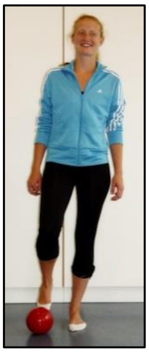

3. Limits of stability: leaning forward, backward or sideward

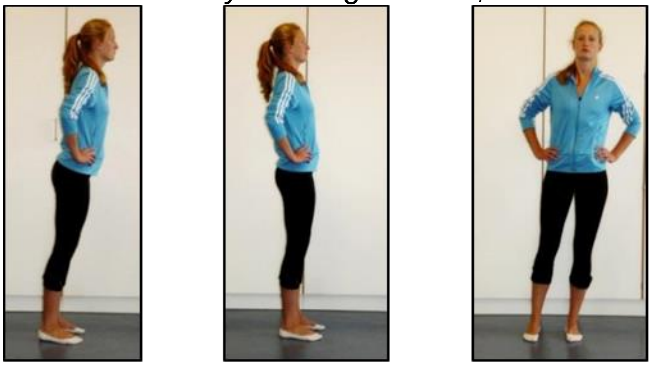

4. Shoulder pulls: participants were perturbed by shoulder pulls from the therapist

Variation / Training progression: In order to reach training progression, exercises 1-4 were performed with the following variations:

- Eyes open/closed
- Look to the side or up to the ceiling
- Participants had to hold a glass of water and focus on it

<u>B) Stance on unstable surfaces</u>

1.

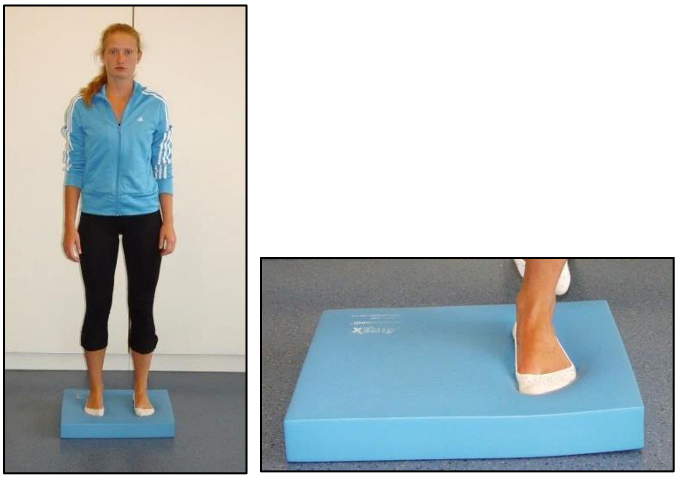

2.

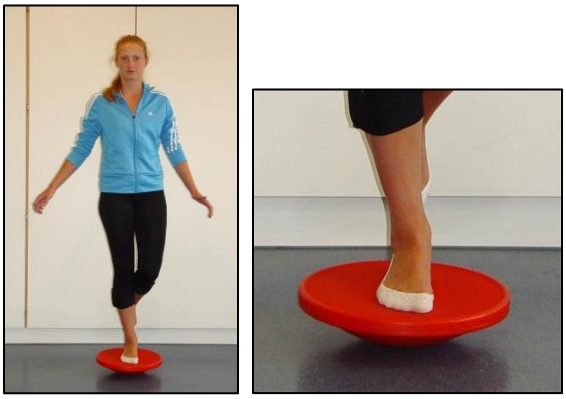

3.

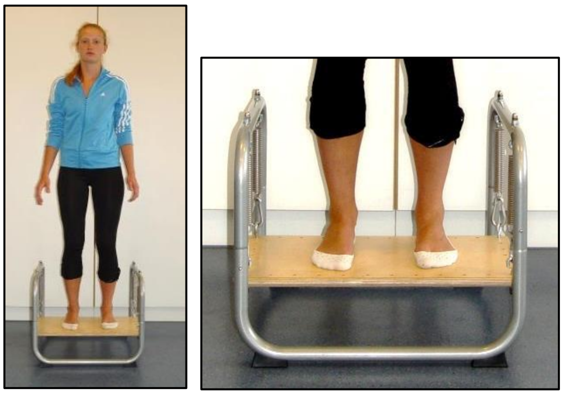

Variation / Training progression: In order to reach training progression, exercises 1-3 were performed with the following variations:

- Eyes open/closed
- Look to the side or up to the ceiling
- Participants had to hold a glass of water and focus on it
- Participants were perturbed by shoulder pulls of the therapist

<u>C) Gait tasks</u>

1.

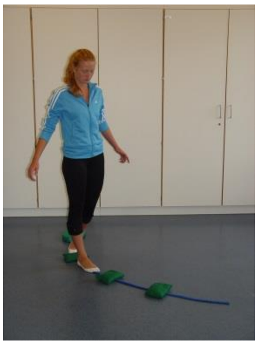 Subjects had to cross different obstacles.

The person of the images gave written consent to publication.
