# Supplementary material for: Resistance versus Balance Training to Improve Postural Control in Parkinson's Disease: A Randomized Rater Blinded Controlled Study
Source: PLoS One. 2015 Oct 26;10(10):e0140584. doi: 10.1371/journal.pone.0140584 (PMC4621054; doi:10.1371/journal.pone.0140584)
Supplement: S3 File — Text which gives further details about the surface perturbations. (PDF) [file pone.0140584.s004.pdf]

Resistance- versus Balance Training to improve postural control in Parkinson's Disease

Christian Schlenstedt[1,2], Steffen Paschen[1], Annika Kruse[2], Jan Raethjen[1], Burkhard Weisser[2], Günther Deuschl[1]

[1] Department of Neurology, Christian-Albrechts-University, Kiel, Germany
[2] Department of Sport Science, Christian-Albrechts-University, Kiel, Germany

Supporting Information 3

Surface Perturbation

Surface perturbations were performed in order to assess feedback- and feedforward postural control under standardized conditions. The subjects were asked to maintain their balance without doing steps while standing on a movable platform which shifted unexpectedly towards anterior- or posterior direction. Participants were not aware neither when the platform would move nor in which direction the surface would change. 20 perturbations were conducted, 10 times in anterior direction ("backward pull") and 10 times in posterior direction ("forward pull"). As in our experience the postural response to a perturbation depends on the previous perturbation type, all subjects received the same randomized order of backward- and forward pulls at baseline and 8-weeks follow-up. The platform was moved 20 cm with a velocity of 0.1 m/s and an acceleration of 10 m/s^2. Participants' balance therefore was perturbed two times - when the surface started to move and when the platform stopped. We used a relatively low velocity and long perturbation time in order to avoid the subjects

doing steps and to let the subjects anticipate the end of the perturbation. The first reaction when the platform started to move can be considered to be mostly feedback controlled. The reaction to the end of the perturbation was feedforward- and feedback controlled. Participants wore a safety harness (without weight assist) to avoid injuries due to falls.

Center of mass (COM) displacement was analyzed by measuring the movement of body segments with an infrared movement analysis system (Qualisys, Gothenburg, Sweden) consisting of six infrared cameras (240 Hz sampling rate). 17 infrared light emitting diodes were placed on anatomic landmarks as described in detail elsewhere [1] and the COM was calculated as the weighted sum of all segments, as adapted from Winter et al. [2]. According to Visser et al. [1] the vector length of three-dimensional COM displacement was calculated. In order to adapt to different biomechanical requirements due to different sizes of subjects, the vector length was normalized to COM height. The average normalized vector length over all backward- and forward pulls was calculated, respectively. The area under the curve of the normalized vector length from the beginning until 1 sec after the perturbation was defined as an instability outcome measure [1]. Trials during which subjects had to take steps were excluded and subjects received two perturbations in each direction before starting the measurement in order to avoid recording first trial reactions [3].

References

1. Visser JE, Allum JH, Carpenter MG, Esselink RA, Speelman JD, et al. (2008) Subthalamic nucleus stimulation and levodopa-resistant postural instability in Parkinson's disease. J Neurol 255: 205-210.
2. Winter DA, Patla AE, Ishac M, Gage WH (2003) Motor mechanisms of balance during quiet standing. J Electromyogr Kinesiol 13: 49-56.
3. Oude Nijhuis LB, Allum JH, Valls-Sole J, Overeem S, Bloem BR (2010) First trial postural reactions to unexpected balance disturbances: a comparison with the acoustic startle reaction. Journal of neurophysiology 104: 2704-2712.
