# Supplementary material for: Resistance versus Balance Training to Improve Postural Control in Parkinson's Disease: A Randomized Rater Blinded Controlled Study
Source: PLoS One. 2015 Oct 26;10(10):e0140584. doi: 10.1371/journal.pone.0140584 (PMC4621054; doi:10.1371/journal.pone.0140584)
Supplement: S4 File — Text which gives further details about the gait analysis. (PDF) [file pone.0140584.s005.pdf]

Resistance- versus Balance Training to improve postural control in Parkinson's Disease

Christian Schlenstedt[1,2], Steffen Paschen[1], Annika Kruse[2], Jan Raethjen[1], Burkhard Weisser[2], Günther Deuschl[1]

[1] Department of Neurology, Christian-Albrechts-University, Kiel, Germany
[2] Department of Sport Science, Christian-Albrechts-University, Kiel, Germany

Supporting Information 4

Gait Analysis

Gait velocity of participants was measured during uninterrupted ground level walking. Subjects were requested to walk 5 times a distance of 5 m at a freely selected comfortable speed. Gait velocity was calculated as the mean of the 5 runs, recorded by light barriers placed at the beginning and at the end of the pathway. Afterwards, subjects were asked to walk 2 min. on a treadmill (length of 2.2 m and width of 0.7 m; Woodway, Weil am Rhein, Germany) with their overground gait velocity. Participants wore a safety harness without weight assist to avoid injuries due to falls. The treadmill comprised two separate belts, each with 4 force transducers (Kistler, Winterthur, Switzerland) (960Hz sampling rate). Contact times (heel strike, toe off) were measured by the force transducers to calculate the following spatio-temporal variables:

- *Mean Stride length:* mean stride time (time from heel strike to heel strike) multiplied by belt speed (cm)[1]

- *Double Support Time:* mean time (ms) both feet equally spend on the ground
- *Stride Time Variability: coefficient of variation of stride time (SD/mean x 100)*
- *Phase Coordination Index (PCI):* Bilateral coordination of gait was assessed by examining the phase between the step timing of the left and right legs [2,3]. In short, each stride defines one gait cycle and the time between the start of a gait cycle and the time point when the other leg's heel strike occurs, was used to determine the phase. Normalizing the step time with respect to the stride time and scaling by 360º, defined the phase of the i-th stride (φi; denoted in degrees). The PCI is a metric that combines the accuracy and consistency of stepping phases generation. The level of accuracy, i.e., how close are the series of generated phases to the value 180º, was assessed by the mean value of the series of absolute differences, $\varphi_i - 180°$, denoted a measure of temporal accuracy as φ_ABS:

  $$\varphi\_ABS = |\varphi_i - 180°|$$

  The degree of consistency of the stepping phase generation was calculated by the coefficient of variation of φ, denoted as φ_CV and given as a percent. Therefore, assuming that Pφ_ABS=100*(φ_ABS/180), PCI was defined according to the following equation:

  $$PCI = \varphi\_CV + P\varphi\_ABS$$

  Thus, PCI is the sum of two relative values, each given as a percentile. Lower PCI values reflect a more consistent and more accurate phase generation and related to different health conditions, with higher values indicating more impaired bilateral coordination of gait [2,4].
- *Asymmetry Index (AI):* |ln(SSWT/LSWT)|, where SSWT and LSWT are the short and long swing time, respectively [5].

References

1. Hoogkamer W, Bruijn SM, Duysens J (2014) Stride length asymmetry in split-belt locomotion. Gait & Posture 39: 652-654.
2. Plotnik M, Giladi N, Hausdorff JM (2008) Bilateral coordination of walking and freezing of gait in Parkinson's disease. Eur J Neurosci 27: 1999-2006.
3. Plotnik M, Giladi N, Hausdorff JM (2007) A new measure for quantifying the bilateral coordination of human gait: effects of aging and Parkinson's disease. Experimental brain research 181: 561-570.
4. Plotnik M, Hausdorff JM (2008) The role of gait rhythmicity and bilateral coordination of stepping in the pathophysiology of freezing of gait in Parkinson's disease. Movement Disorders 23: 444-450.
5. Plotnik M, Giladi N, Balash Y, Peretz C, Hausdorff JM (2005) Is freezing of gait in Parkinson's disease related to asymmetric motor function? Ann Neurol 57: 656-663.
