# Supplementary material for: Resistance versus Balance Training to Improve Postural Control in Parkinson's Disease: A Randomized Rater Blinded Controlled Study
Source: PLoS One. 2015 Oct 26;10(10):e0140584. doi: 10.1371/journal.pone.0140584 (PMC4621054; doi:10.1371/journal.pone.0140584)
Supplement: S5 File — Text which gives further details about the strength testing. (PDF) [file pone.0140584.s006.pdf]

Resistance- versus Balance Training to improve postural control in Parkinson’s Disease

Christian Schlenstedt[1,2], Steffen Paschen[1], Annika Kruse[2], Jan Raethjen[1], Burkhard Weisser[2], Günther Deuschl[1]

[1] Department of Neurology, Christian-Albrechts-University, Kiel, Germany
[2] Department of Sport Science, Christian-Albrechts-University, Kiel, Germany

Supporting Information 5

Strength Testing

A warm-up period of 5 minutes on a bicycle ergometer at 40-70W was mandatory for each subject before strength testing. Maximal isometric leg strength was measured on a custom designed leg press equipped with a force platform (Kistler, Winterthur, Switzerland) (1000Hz sampling rate). The subjects were positioned with the hip, knee and ankle angles adjusted at 90°. The position of each subject was documented that it was identical under the baseline-, 8- and 12-weeks follow-up conditions. The subjects were allowed to stabilize their upper body by holding on to handles attached to the leg press. Two submaximal isometric contractions were allowed to get accustomed to the testing procedure. Thereafter, each subject performed three leg press exercises with each leg separately with maximal voluntary effort. Subjects were carefully instructed to contract “as fast and forcefully as possible” and to hold the maximal contraction for 3 sec. A 2 minutes rest was given between the trials of one leg and a 5 minutes rest was given between the 3 trials of the left/right leg. Maximal

voluntary contraction (MVC) was defined as the highest value of the 3 attempts, for each leg respectively. As described elsewhere the trial with the highest MVC was analyzed during later offline analysis [1]. The force signal was filtered by a digital fourth order recursive Butterworth low-pass filter, using a cutoff frequency of 50 Hz. The onset of muscle contraction was defined as the time at which the force curve exceeded baseline force by 15 N [2]. Peak rate of force development (RFD) was defined as the maximal slope of the force time curve (∆force/∆time). Additionally, submaximal RFD-values were calculated as mean slope of the force-time curve (d F/d t) over time intervals of 0–100, 0-200, 0-300 and 0-500ms relative to the onset of force [1,2,3].

MVC and RFD were analyzed for the more- and less-affected Parkinson's Disease side separately – defined by comparing the sums of the UPDRS items 20-26 for the left and right side separately [4,5].

References

1. Gruber M, Gollhofer A (2004) Impact of sensorimotor training on the rate of force development and neural activation. Eur J Appl Physiol 92: 98-105.
2. Maffiuletti NA, Bizzini M, Widler K, Munzinger U (2010) Asymmetry in quadriceps rate of force development as a functional outcome measure in TKA. Clinical orthopaedics and related research 468: 191-198.
3. Aagaard P, Simonsen EB, Andersen JL, Magnusson P, Dyhre-Poulsen P (2002) Increased rate of force development and neural drive of human skeletal muscle following resistance training. J Appl Physiol 93: 1318-1326.
4. Geurts AC, Boonstra TA, Voermans NC, Diender MG, Weerdesteyn V, et al. (2011) Assessment of postural asymmetry in mild to moderate Parkinson's disease. Gait & Posture 33: 143-145.
5. Plotnik M, Giladi N, Hausdorff JM (2008) Bilateral coordination of walking and freezing of gait in Parkinson's disease. Eur J Neurosci 27: 1999-2006.
