# Supplementary material for: Resistance versus Balance Training to Improve Postural Control in Parkinson's Disease: A Randomized Rater Blinded Controlled Study
Source: PLoS One. 2015 Oct 26;10(10):e0140584. doi: 10.1371/journal.pone.0140584 (PMC4621054; doi:10.1371/journal.pone.0140584)
Supplement: S1 Protocol — (PDF) [file pone.0140584.s007.pdf]

Study protocol

(English translation of the german study protocol; for the complete study protocol see german version)

Resistance versus Balance Training to improve postural control in Parkinson's disease

Responsible:

Prof. Dr. G. Deuschl / C. Schlenstedt / Dr. J. Raethjen

Department of Neurology

University Hospital Schleswig-Holstein

Campus Kiel

Schittenhelmstrasse 10

24105 Kiel

Germany

Background

Postural Instability is one of the major symptoms of patients with Parkinson’s disease (PD). Postural instability, reduced muscle strength and Freezing of gait are independent risk factors for falls. Postural control can only hardly be improved by medication or deep brain stimulation in individuals with Parkinson’s disease. Exercise training therefore plays an important role in the treatment of this symptom.

Only few studies analyzed the effects of resistance training in patients with Parkinson’s disease. Especially the impact of this training type in postural control still remains unclear. Although largely performed in physical therapy, the effects of isolated balance training are rarely analyzed.

Objective

The objective of this study is to compare resistance training with balance training to improve postural control in individuals with Parkinson’s disease.

Study design

The study is expected to enroll 40 patients; they will be randomized into one of the following groups:

Group1. 20 patients (resistance training)

Group 2. 20 patients (balance training)

The two groups will submit to the following programs:

Group 1: Resistance training with the aim to improve muscle strengths of the lower limbs. The trained muscle groups will be hip flexors, extensors and abductors, knee flexors and extensors, ankle dorsiflexors and plantarflexors. The training will be conducted 60 minutes, two times per week for eight weeks.

Group 2: Balance training will involve stance- and gait tasks which require feedforward and feedback postural control. Subjects will train on stable and unstable surfaces. The training will be conducted 60 minutes, two times per week for eight weeks.

Participants

Subjects will be recruited from the department of Neurology and the department of Physical Therapy (University Hospital Schleswig-Holstein, Campus Kiel) and the Parkinson’s disease support group Kiel. Inclusion criteria are the diagnosis of idiopathic Parkinson’s disease and the presence of postural instability. Exclusion criteria are cardiopulmonary/metabolic diseases that could interfere with the safe conduct of the study protocol, unstable medication, severe cognitive impairments that do not allow the patients to follow exercise instructions and participation in a resistance or balance training program (beside usual physical therapy).

Randomization

Participants will be randomized in matched pairs for gender and level of postural instability into the resistance and balance training group.

Training

Training will be performed in the Physical Therapy areas of the Department of Neurology, the Department of Physical Therapy and the Department of Sports Science, Christian-Albrechts-University Kiel. Each training session will last 60 minutes. C. Schlenstedt (sports scientist) will lead the training sessions with the help of a sports student/student of physical therapy.

Safety issues of the training

A risk of injury can never be totally eliminated when performing exercises. The risk of injury will be minimized by the following: Each training session will start with a warm-up to avoid injuries of the musculoskeletal system. Each patient will be asked about any complication to avoid overload. The training protocol was designed with regard to the standard training principles and with respect to the age and the disease of the subjects.

Measurements

To analyze the efficacy of the training types, subjects will be assessed at baseline, 8-weeks follow-up and 12-weeks follow-up. Patients will be tested in the medication “on” state. The following outcome measures will be conducted:

- Berg Balance Scale [note of the authors: with approval of the ethics committee we used the Fullerton Advanced Balance Scale instead of the Berg Balance Scale]

- Falls diaries
- Strength testing: leg strength will be measured in a custom designed leg press to assess maximal voluntary contraction and rate of force development
- Analysis of Center of Mass displacement by measuring the movement of body segments with an infrared movement analysis system during surface perturbation: the subjects will be asked to maintain their balance without doing steps while standing on a movable platform which shifts unexpectedly towards anterior- or posterior direction
- Gait analysis: gait velocity will be measured on overground, gait analysis will be performed on a motor-driven treadmill with the overground gait velocity
- Unified Parkinson’s Disease Rating Scale
- Patients with Freezing of Gait: Freezing score of Ziegler et al. (2010) and Freezing of Gait Questionnaire
- Center of pressure displacement during gait initiation
- Parkinson’s Disease Questionnaire (PDQ-39): to assess quality of life

All clinical tests will be videotaped and rated by a second rater, blinded to group allocation and assessment time.

STUDY OUTLINE

**Title**	**Resistance versus Balance Training to improve postural control in Parkinson's disease**
**Responsible**	Prof. Dr. G. Deuschl / C. Schlenstedt / Dr. J. Raethjen<br>Department of neurology<br>UK-SH, Campus Kiel<br>Schittenhelmstrasse 10<br>24105 Kiel<br>Germany
**Objective**	To compare resistance training with balance training to improve postural control in individuals with Parkinson's disease
**Design**	Randomized rater blinded controlled trial
**Subjects**	40 individuals with idiopathic Parkinson's disease
**Intervention**	Group 1 (n=20): resistance training<br><br>Group 2 (n=20): balance training
**Assessments**	**1** baseline<br>**2** 8 weeks follow-up<br>**3** 12 weeks follow-up
**Outcome measures**	- Berg Balance Scale [changed to Fullerton Advanced Balance Scale with the approval of the ethics committee]<br>- Falls diaries<br>- Strength testing<br>- Center of Mass analysis during surface perturbation<br>- Gait analysis<br>- UPDRS<br>- Freezing Score<br>- Freezing of Gait Questionnaire<br>- Analysis of gait initiation<br>- PDQ-39
