# Supplementary material for: Resistance versus Balance Training to Improve Postural Control in Parkinson's Disease: A Randomized Rater Blinded Controlled Study
Source: PLoS One. 2015 Oct 26;10(10):e0140584. doi: 10.1371/journal.pone.0140584 (PMC4621054; doi:10.1371/journal.pone.0140584)
Supplement: S2 Protocol — (PDF) [file pone.0140584.s008.pdf]

Projektbeschreibung zur Vorlage bei der Ethikkommission der Medizinischen Fakultät der Universität Kiel

Trainierbarkeit der posturalen Kontrolle bei Parkinsonpatienten

Projektleiter: Prof. Dr. G. Deuschl, C. Schlenstedt, Dr. J. Raethjen

Durchführende: C. Schlenstedt, Dr. J. Raethjen, Physiotherapeut/Sportstudent

Institution: Klinik für Neurologie, UK-SH, Campus Kiel

Wissenschaftlicher Hintergrund:

Posturale Instabilität zählt neben Tremor, Rigor und Bradykinesie bis hin zur Akinese zu den Kardinalsymptomen der Parkinsonkrankheit. Hinsichtlich des Sturzrisikos gelten posturale Instabilität, muskuläre Defizite sowie Freezing als unabhängige Risikofaktoren (Latt 2006). Parkinsonpatienten stürzen durchschnittlich doppelt so häufig im Vergleich zu Gesunden (Bloem et al. 2001). 27% der Patienten stürzen mindestens einmal pro Monat, 15% sogar mindestens einmal pro Woche (Latt 2006). In Anbetracht dieses hohen Sturzrisikos gilt es Wege zu finden, die posturale Stabilität und die Kraftfähigkeiten bei Parkinsonpatienten zu verbessern, um Risikofaktoren zu reduzieren. Die medikamentöse Behandlung führt bisher zu keiner zufrieden stellenden Verbesserung der posturalen Kontrolle (Bloem et al. 1996; Horak et al. 1996; Frank et al. 2000), wodurch der bewegungstherapeutische Ansatz als Behandlungsmethode an Stellenwert gewinnt.

Es konnte gezeigt werden, dass sich durch Sensomotorisches Training (SMT) die posturale Kontrolle bei Parkinsonpatienten verbessern lässt und sich dadurch das Sturzrisiko reduziert (Smania et al. 2010). Ob sich durch SMT die Explosivkraft bei Parkinsonpatienten steigern lässt, wie dies bei gesunden älteren Menschen gezeigt wurde (Gruber und Gollhofer 2004; Granacher et al. 2007; Gruber et al. 2007), ist bisher ungeklärt. Es konnte nachgewiesen werden, dass sich durch Krafttraining die Maximalkraft und die Mobilität bei Parkinsonpatienten steigern lässt (Scandalis et al. 2001; Hirsch et al. 2003; Dibble et al. 2006; Schilling et al. 2010). Die Auswirkungen von Krafttraining auf die Sturzhäufigkeit sowie die posturale Kontrolle bleiben hierbei ungeklärt.

Das beantragte Projekt beinhaltet zwei Schwerpunkte: Zum Einen soll die Effektivität von SMT und Krafttraining bei Parkinsonpatienten verglichen werden. Dabei geht es um die Frage, welche dieser Trainingsformen sich am besten zur Verbesserung des Sturzrisikos, der posturalen Kontrolle sowie der Kraftfähigkeiten eignet und in welchem Maße die Trainingsformen in der Therapie Anwendung finden sollten. Zweitens soll untersucht werden, wie sich die möglichen Leistungsverbesserungen durch SMT zwischen Parkinsonpatienten und gesunden älteren Menschen unterscheiden. Dabei geht es um die Frage, ob Parkinsonpatienten über eine ähnliche motorische Reserve verfügen wie Gesunde.

Studiendurchführung:

Es sollen zwei Parkinsongruppen und eine Gruppe mit gesunden älteren Menschen an der Studie teilnehmen. Eine Parkinsongruppe wird SMT und die andere Parkinsongruppe Krafttraining durchführen. Die Gruppe der Gesunden wird SMT durchführen. Dadurch können die zwei Trainingsformen (SMT vs. Krafttraining) bei Parkinsonpatienten sowie die motorische Reserve zwischen Parkinsonpatienten und Gesunden bezüglich SMT verglichen werden. Die Testparameter sollen vor und nach der Intervention sowie vier Wochen im Follow-up erhoben werden.

Gruppen:	P1:	Parkinsonpatienten – SMT
	P2:	Parkinsonpatienten – Krafttraining
	G3:	Gesunde Probanden – SMT

Probandenrekrutierung:

Es sollen 40 Parkinsonpatienten und 20 gesunde ältere Menschen an der Studie teilnehmen (P1: n=20; P2: n=20; G3: n=20). Die Parkinsonpatienten sollen über die Datenbank der Klinik für Neurologie, die Parkinsonselbsthilfegruppe, stationäre und ambulante Patienten der Klinik für Neurologie sowie ambulante Patienten der Physiotherapie rekrutiert werden. Die gesunden Probanden sollten möglichst durch die Ehepartner der Parkinsonpatienten gebildet werden. Die Geschlechterverteilung soll im Vergleich der Gruppen gleich sein. Allerdings sind aus sportwissenschaftlicher Sicht hinsichtlich der Adaptationsprozesse an das Training generell keine geschlechtsspezifischen Unterschiede zu erwarten.

Einschlusskriterien:

Parkinsonpatienten:

- Diagnose: klinisch gesicherter Morbus Parkinson
- Hoehn & Yahr Stadium: 2 – 4
- die Patienten sollen in der Lage sein, ohne Rollator zu gehen und ohne Hilfe min. 2 Min. sicher zu stehen
- posturale Instabilität

Gesunde:

- die Probanden sollen in der Lage sein ohne Rollator zu gehen und ohne Hilfe min. 2 Min. sicher zu stehen
- Alter: 60 – 80 Jahre

Ausschlusskriterien:

Parkinsonpatienten / Gesunde:

- Teilnahme an einem SMT oder Krafttraining (neben der normalen Physiotherapie) in dem Zeitraum 3 Monate vor dem Interventionsbeginn
- Es gelten die üblichen Kontraindikationen für Sport im Alter (Denk und Belinová 2003) wie z.B.: kardiovaskuläre Erkankungen, Organerkrankungen die eine körperliche Belastung ausschließen (z.B. Leber, Niere), höhergradige unbehandelte Hypertonie, etc.
- schwere kognitive Störungen
- bei Parkinsonpatienten: völlig unzureichende Wirkung der dopaminergen Medikation

Randomisierung:

Die Parkinsonpatienten sollen extern in zwei gleich große (20:20) Gruppen randomisiert werden. Dafür sollen gematchte Paare gebildet werden, wobei ein Proband eines Paares nach dem Zufallsprinzip am SMT teilnehmen soll und der andere Proband am Krafttraining. Die Probanden sollen hinsichtlich Alter, Geschlecht und Wert des Berg Balance Scales gematcht werden. Dadurch soll gewährleistet werden, dass zwei im Bezug zum Alter, Geschlecht und Gleichgewichtsfähigkeit vergleichbare Gruppen gebildet werden.

Training:

Das Training soll im Gymnastikraum des Zentrums für Physiotherapie und physikalische Therapie, im Gymnastikraum des Neurozentrums sowie im

Trainingsraum des Instituts für Sport und Sportwissenschaft der Universität Kiel stattfinden. Es soll in Kleingruppen mit 6 – 8 Personen über eine Stunde trainiert werden. C. Schlenstedt wird als Sportwissenschaftler hauptsächlich die Leitung der Trainingsstunden durchführen. Ergänzend wird ein Sportstudent/Physiotherapieschüler bei der Trainingsdurchführung anwesend sein.

Bei dem SMT handelt es sich um Gleichgewichtsübungen auf verschiedenen instabilen Unterlagen. Parkinsonpatienten und Gesunde führen (angepasst an das individuelle Leistungsniveau) das gleiche SMT durch. Das Krafttraining wird nicht an Kraftgeräten sondern mit freien Gewichten und Kleingeräten wie elastische Übungsbänder durchgeführt.

Sicherheitsaspekte beim Training:

Ein Verletzungsrisiko kann bei einem sportlichen Training nie generell ausgeschlossen werden. Dies soll jedoch durch folgende Kriterien minimiert werden: Bei beiden Übungsformen wird ein Aufwärmtraining durchgeführt um Verletzungen des Bewegungsapparates zu vermeiden. Der Übungsleiter wird die Teilnehmer beim Training auf mögliche Komplikationen befragen, um eine Überbelastung oder sonstige Schädigungen frühzeitig zu erkennen. Der Trainingsplan wurde nach den üblichen Prinzipien der Methodik der Trainingslehre aufgebaut und auf das entsprechende Probandenkollektiv zugeschnitten. Die Übungsformen fanden zum Teil bei anderen Studien Anwendung (Hirsch et al. 2003; Granacher et al. 2006; Granacher et al. 2007; Granacher et al. 2009; Smania et al. 2010) und die Durchführung erwies sich insgesamt als unproblematisch. Vor allem beim SMT werden die Übungen mit ausreichender Hilfeleistung, bzw. Möglichkeit zum Festhalten durchgeführt. Ein genauer Trainingsentwurf des SMT und des Krafttrainings befindet sich im Anhang.

Untersuchungszeitpunkte und Testverfahren:

Zur Bestimmung der Trainingseffekte sollen die Teilnehmer direkt vor dem Trainingszeitraum, direkt nach Beendigung des Übungszeitraumes sowie 4 Wochen im Follow-up untersucht werden. Die Untersuchungen sollen im „ON“ 1,5 Stunden nach Medikamenteneinnahme stattfinden. Die Messungen sollen im Motoriklabor der Neurologie durchgeführt werden. Dabei sollen folgende Tests durchgeführt werden:

- Berg Balance Scale: klinischer Gleichgewichtstest (Qutubuddin et al. 2005)

- Sturzkalender: Die Teilnehmer sollen über einen Sturzkalender die Sturzhäufigkeit über einen Monat dokumentieren (die Sturzkalender werden einen Monat vor dem jeweiligen Untersuchungszeitpunkt verteilt)
- Die Explosiv- und Maximalkraft wird über eine Kraftmessplatte in der Beinpresse bei isometrischer Muskelkontraktion bestimmt.
- Dynamische Posturographie: Bestimmung der posturalen Reaktion (durch Körperschwerpunktbestimmung) bei translatorischen Störreizen beim Stand: Die Teilnehmer stehen hierfür auf dem Laufband. Die Standfläche soll über das Laufband translatorisch versetzt werden und die Probanden sollen versuchen ihr Gleichgewicht zu halten. Die Störreize sollen randomisiert nach anterior und posterior ausgelöst werden. Dabei soll der Schwankungsweg des Körpers über ein Infrarot-Marker-System bestimmt werden.
- Ganganalyse: Es sollen die Schrittlänge, die Zeit der Doppelstandphase sowie die Schrittfrequenz beim Gang auf einem Laufband bestimmt werden. Hierfür soll eine kinematische Analyse des Gangbildes durchgeführt werden, wofür den Probanden Marker auf die Haut geklebt werden müssen (siehe dynamische Posturographie).
- UPDRS: Unified Parkinson’s Disease Rating Scale Teil 2 und 3
- Bei Patienten mit Freezingphänomen: Zur Bestimmung des Freezing-Vorkommens soll der Freezing-Score von Ziegler et al. (2010) durchgeführt werden: Dabei wird die Häufigkeit von Freezingepisoden bei bestimmten alltäglichen Bewegungen (Aufstehen, 360°-Drehung, Passieren von Türrahmen) gemessen.
- Bei Patienten mit Freezingphänomen: FOGQ (Freezing of gait questionnaire): Fragebogen zur Bestimmung der Häufigkeit von Freezingepisoden (Giladi et al. 2009)
- Ganginitiierung: Es sollen die Massendruckpunkt- (Center of Pressure) und Körperschwerpunkt-Verläufe bei der Ganginitiierung bestimmt werden. Der Massendruckpunkt soll dabei über eine Kraftmessplatte und der Körperschwerpunkt über eine kinematische Markeranalyse (siehe dynamische Posturographie) bestimmt werden.
- PDQ-39: krankheitsspezifischer Fragebogen zur Erfassung der Lebensqualität (Jenkinson et al. 1997; Berger et al. 1999)

Der Berg Balance Scale, UPDRS sowie der Freezing Score sollen als klinische Tests rater-geblindet ausgewertet werden.

Sicherheitsaspekte der Untersuchungsverfahren:

Alle in dieser Studie geplanten Untersuchungsmethoden sind nichtinvasiv. Die Fragebögen (Sturzkalender, FOGQ, PDQ-39) sind unproblematisch. Die klinischen Testverfahren (Berg Balance Scale, UPDRS, Freezing-Score) sind Standartuntersuchungsverfahren, bei welchen eine Schädigung des Patienten äußerst unwahrscheinlich ist. Die biomechanischen Untersuchungsmethoden (Explosivkraft- und Maximalkraftmessung, dynamische Posturographie, Ganganalyse, Ganginitiierung) sollen im Folgenden genauer beschrieben werden:
Bei der *Explosiv- und Maximalkraftmessung* wird vor der Untersuchung ein Aufwärmprogramm durchgeführt. Durch ein Aufwärmen sollen Verletzungen des Bewegungsapparates auch bei maximalem Kraftaufbau verhindert werden. Die Kraftmessung in der Beinpresse ist ein Routineverfahren in der sportlichen Leistungsdiagnose und wurde in anderen Studien auch bei älteren unsportlichen Menschen angewendet (Granacher et al. 2007).
Bei der *dynamischen Posturographie* wird nicht darauf abgezielt durch den translatorischen Störreiz (Verschieben der Standfläche durch das Laufband) einen Sturz zu provozieren. Dementsprechend soll das Laufband mit einer Geschwindigkeit angesteuert werden, bei der die Probanden in der Lage sind ihr Gleichgewicht zu halten. Zudem die Probanden tragen einen Sicherheitsgurt, welcher sie im Fall des Gleichgewichtsverlustes vor einem Sturz bewahrt. Der Sicherheitsgurt ist an der Decke mit einem Notschalter verbunden, wodurch bei Belastung des Gurtes das Laufband automatisch gestoppt wird. Ein weiterer Notschalter befindet sich in unmittelbarer Reichweite des Untersuchungsdurchführenden.
Die *Ganganalyse* wird auf dem Laufband durchgeführt. Dabei wird das Laufband mit der individuellen Ganggeschwindigkeit (welche vorher auf normalem Untergrund gemessen wird) betrieben. Auch bei der Ganganalyse tragen die Probanden den Sicherheitsgurt. In der Neurologie besteht reichlich Erfahrung im Umgang mit der Ganganalyse auf einem Laufband.
Bei der *Ganginitiierung* handelt es sich um die Messung des Standes auf einer Kraftmessplatte mit anschließender Durchführung eines normalen Schrittes.

Bei der *dynamischen Posturographie, der Ganganalyse und der Ganginitiierung* sollen die Bewegungen der Probanden analysiert werden. Hierfür müssen Marker (Durchmesser ca. 18mm) auf die Extremitäten der Probanden geklebt werden, welche über Infrarotkameras registriert werden. Die runden Marker werden mit Klebeband auf die Haut der Probanden geklebt. Die geklebten Marker können für alle drei Testverfahren verwendet werden.

Prof. Dr. G. Deuschl C. Schlenstedt

Anlagen:

- Trainingsablauf Sensomotorisches Training
- Trainingsablauf Krafttraining
- Informationsblatt zur Studie
- Information und Aufklärung zur Studie
- Einverständniserklärung

Literaturverzeichnis

Berger, K., Broll, S., Winkelmann, J., Heberlein, I., Muller, T., Ries,V. (1999). *Untersuchung zur Reliabilität der deutschen Version des PDQ-39: Ein krankheitsspezifischer Fragebogen zur Erfassung der Lebensqualität von Parkinsonpatienten* Stuttgart: Thieme.

Bloem, B. R., Beckley, D. J., van Dijk, J. G., Zwinderman, A. H., Remler, M. P.,Roos, R. A. (1996). Influence of dopaminergic medication on automatic postural responses and balance impairment in Parkinson's disease. *Mov Disord* 11(5): 509-521.

Bloem, B. R., Grimbergen, Y. A., Cramer, M., Willemsen, M.,Zwinderman, A. H. (2001). Prospective assessment of falls in Parkinson's disease. *J Neurol* 248(11): 950-958.

Denk, H.,Belinová, K. (2003). *Handbuch Alterssport*. Schorndorf: Hofmann.

Dibble, L. E., Hale, T., Marcus, R. L., Gerber, J. P.,Lastayo, P. C. (2006). The safety and feasibility of high-force eccentric resistance exercise in persons with Parkinson's disease. *Arch Phys Med Rehabil* 87(9): 1280-1282.

Frank, J. S., Horak, F. B.,Nutt, J. (2000). Centrally initiated postural adjustments in parkinsonian patients on and off levodopa. *J Neurophysiol* 84(5): 2440-2448.

Giladi, N., Tal, J., Azulay, T., Rascol, O., Brooks, D. J., Melamed, E., Oertel, W., Poewe, W. H., Stocchi, F.,Tolosa, E. (2009). Validation of the freezing of gait questionnaire in patients with Parkinson's disease. *Mov Disord* 24(5): 655-661.

Granacher, U., Gollhofer, A.,Strass, D. (2006). Training induced adaptations in characteristics of postural reflexes in elderly men. *Gait Posture* 24(4): 459-466.

Granacher, U., Gruber, M.,Gollhofer, A. (2009). Auswirkungen von sensomotorischem Training auf die posturale Kontrolle älterer Männer. *Deutsche Zeitschrift für Sportmedizin* 60(12): 387-392.

Granacher, U., Gruber, M., Strass, D.,Gollhofer, A. (2007). Auswirkungen von sensomotorischem Training im Alter auf die Maximal- und Explosivkraft. *Deutsche Zeitschrift für Sportmedizin* 58(12): 446-451.

Gruber, M.,Gollhofer, A. (2004). Impact of sensorimotor training on the rate of force development and neural activation. *Eur J Appl Physiol* 92(1-2): 98-105.

Gruber, M., Gruber, S. B., Taube, W., Schubert, M., Beck, S. C.,Gollhofer, A. (2007). Differential effects of ballistic versus sensorimotor training on rate of force development and neural activation in humans. *Journal of strength and conditioning research / National Strength & Conditioning Association* 21(1): 274-282.

Hirsch, M. A., Toole, T., Maitland, C. G.,Rider, R. A. (2003). The effects of balance training and high-intensity resistance training on persons with idiopathic Parkinson's disease. *Arch Phys Med Rehabil* 84(8): 1109-1117.

Horak, F. B., Frank, J.,Nutt, J. (1996). Effects of dopamine on postural control in parkinsonian subjects: scaling, set, and tone. *J Neurophysiol* 75(6): 2380-2396.

Jenkinson, C., Fitzpatrick, R., Peto, V., Greenhall, R.,Hyman, N. (1997). The Parkinson's Disease Questionnaire (PDQ-39): development and validation of a Parkinson's disease summary index score. *Age Ageing* 26(5): 353-357.

Latt, M. D. (2006). Why do people with Parkinson's Disease fall? Faculty of Medicine. Sydney, University of Sydney. PhD thesis.

Qutubuddin, A. A., Pegg, P. O., Cifu, D. X., Brown, R., McNamee, S.,Carne, W. (2005). Validating the Berg Balance Scale for patients with Parkinson's disease: a key to rehabilitation evaluation. *Arch Phys Med Rehabil* 86(4): 789-792.

Scandalis, T. A., Bosak, A., Berliner, J. C., Helman, L. L.,Wells, M. R. (2001). Resistance training and gait function in patients with Parkinson's disease. *Am J Phys Med Rehabil* 80(1): 38-43; quiz 44-36.

Schilling, B. K., Pfeiffer, R. F., Ledoux, M. S., Karlage, R. E., Bloomer, R. J.,Falvo, M. J. (2010). Effects of moderate-volume, high-load lower-body resistance training on strength and function in persons with Parkinson's disease: a pilot study. *Parkinson's disease* 2010: 824734.

Smania, N., Corato, E., Tinazzi, M., Stanzani, C., Fiaschi, A., Girardi, P.,Gandolfi, M. (2010). Effect of balance training on postural instability in patients with idiopathic Parkinson's disease. *Neurorehabil Neural Repair* 24(9): 826-834.

Ziegler, K., Schroeteler, F., Ceballos-Baumann, A. O.,Fietzek, U. M. (2010). A new rating instrument to assess festination and freezing gait in Parkinsonian patients. *Mov Disord* 25(8): 1012-1018.

<u>Trainingsablauf Sensomotorisches Training (SMT):</u>

Hilfsmittel: dünne Matten, Airex-Matten, Therapiekreisel, Schaukelbrett, Posturomed, Minitrampolin, Weichbodenmatte

1. Aufwärmphase (10 Min.):
    - Leichte Herz-Kreislauf-Aktivierung und Mobilitsation: z.B. Gehen, Armkreisen, Körperstreckung, etc.
    - Schulung des *kurzen Fußes nach Janda*

2. Statisches SMT (30 Min.):
    - Fester Untergrund (2 der folgenden Übungen, 20 Sec. Aktivität, 40 Sek. Pause, 4 Wiederholungen, danach eine längere Pause von 3 Minuten; Durchführung im Stand mit ca. 30° gebeugten Knien, Oberkörper aufrecht) :
        - bipedaler Stand: Körperschwerpunkt (KSP)-Verlagerung von einem Bein auf das Andere (evtl. zwei Personenwaagen), vom Vorfuß auf Ferse (KSP soll an den Rand von der Unterstützungsfläche gebracht werden
        - bipedaler Stand: Augen schließen / Blick nach oben / Blick nach hinten über die Schulter
        - monopedaler Stand: Punkt fixieren / Blick zur Seite / Arme zur Seite gestreckt / Arme in Hüfte gestützt / Blick zu geschlossenen Händen / Becher Wasser halten
        - bipedaler Stand: Blick nach vorne: Perturbationsreize (Schubsen an der Hüfte/Schulter)
        - bipedaler Stand auf einer Schrägen (Brett): Blick zur Seite / nach oben
        - Schrittstellung: Blick nach oben / zur Seite, Augen offen / geschlossen

    - Instabiler Untergrund (bipedaler Stand (monopedal für Fortgeschrittene) (2 der folgenden Übungen, 20 Sec. Aktivität, 40 Sek. Pause, 4 Wiederholungen, danach eine längere Pause von 3 Minuten):
        - ruhiger Stand auf:
            - Dünne Matte / Airex Matte / Weichbodenmatte
            - Posturomed
            - Minitrampolin
            - Federschaukel
            - Therapiekreisel

3. Dynamisches SMT (20 Min.) (2 der folgenden Übungen mit 3 Wiederholungen und 1 Min. Pause):
    - Gewichtsverlagerung von einem Bein auf das Andere (Pendeln), dann auf der Stelle gehen, dann auf der Stelle umdrehen (so wenig Schritte wie möglich)
    - Gehen / Umdrehen auf Weichbodenmatte

- Auf- und absteigen: Airex-Matte / Weichbodenmatte / Federschaukel / Posturomed / Schräge (Parcours)
- Gehen mit Perturbationsreizen (Schubsen): in vorgegebenen Bahn bleiben; Variation: mit geschlossenen Augen
- Gehen mit Objekt balancieren
- Über Seil (auf dem Boden liegend) balancieren

Trainingsablauf Krafttraining:

Bei der Durchführung des Krafttrainings ist auf folgende Aspekte zu achten:

- *Ausatmen = Muskeln anspannen*
- *Einatmen = Muskeln lösen und entspannen*
- *Pressatmung sollte vermieden werden, bei allen Übungen ruhig und gleichmäßig atmen*
- *Am besten: durch die Nase einatmen und durch den Mund ausatmen*
- *Bei Übungen im Stand: in den Knien leicht beugen (Beine nicht durchstrecken!) um ein Hohlkreuz zu vermeiden*
- *Intensität: 60-80% der Maximalkraft, sodass nach 8-12 Wdh. eine vollständige Ermüdung eintritt*
- *Vollständige Pause zwischen den Übungen*

Hilfsmittel: Matten, Stühle, elastische Bänder, Handtücher

1. Aufwärmphase (10 Min.):
    - Leichte Herz-Kreislauf-Aktivierung und Mobilitsation: z.B. Gehen, Armkreisen, Körperstreckung, etc.
    - Stabiler Stand: Im stand die Füße hüftbreit geöffnet (parallel), Hände an den Hüftknochen, dann langsam die Knie beugen und das Becken vorschieben, Po anspannen, Bauch einziehen – lösen und langsam die Beine strecken
2. Kräftigungsphase (40 Min.):
    - Von den 6 nachfolgenden Muskelgruppen (Plantarflexoren, Dorsalextensoren, Ischiocrurale Muskulatur, Knieextensoren, Hüftbeuger, Hüftabduktoren) werden pro Trainingsstunde 5 trainiert. Dabei wird pro Muskelgruppe eine Übung mit 3 Sätzen und 8-12 Wdh. durchgeführt.
3. Lockerungsphase (10 Min.):
    - Die beteiligte Muskulatur soll in dieser Phase gelockert und gedehnt werden.

Beteiligte Muskelgruppen:

Plantarflexoren (Sprunggelenk):

- Die Teilnehmer sitzen auf einem Stuhl und sind nach vorne gebeugt (aufstützen der Arme auf Oberschenkel): kontrolliertes Heben der Fersen in den Ballenstand, dann langsames Senken und dann Hochziehen der Fußspitzen zum Fersenstand
- Steigerung: wie oben, aber im Stand mit Festhalten (Sprossenwand/Stuhl oder mit Partner gegenüber): beidbeinig oder einbeinig; Variation: Im Stand auf einer Stufe oder Sprossenwand
- Mit elastischem Band: das Band wird um die Fußspitze eines Beines gelegt und mit beiden Händen festgehalten, nun den Fuß strecken und wieder lösen

Dorsalextensoren (Sprunggelenk):

- Mit elastischem Band: Partner sitzen sich im Langsitz gegenüber, Hande stützen hinter dem Rücken, das Band wird um die gegenüberliegenden Füße gebunden (Abstand der Füße ca. 20cm): nun beide Fußspitzen gleichzeitig Richtung Knie anziehen; die Übung kann auch alleine mit einer Sprossenwand durchgeführt werden

Ischiocrurale Muskulatur:

- Die Teilnehmer liegen auf dem Bauch auf einem Stuhl/Kasten, die Beine sind mit 90° (Hüfte/Knie) gebeugt: wechselseitiges Beinstrecken
- Rückenlage mit angewinkelten Beinen, evtl. Handtuch unter den Kopf und Arme zur Seite legen, Handflächen auf den Boden: beidbeiniges Beckenheben bis Rumpf und Oberschenkel eine Gerade bilden, dann wieder langsames Absenken des Beckens in Ausgangsposition; evtl.: Ausatmen=Lendenwirbelsäule gegen den Boden drücken und das Becken nach vorne schieben, Po anspannen
  Einatmen=Langsam das Becken senken
  (Steigerung: einbeiniges Beckenheben (anderes Bein gestreckt); Fußspitzen anheben; Füße auf Stuhl absetzen und dann Becken anheben)
- Im Kniestand (Knie geöffnet) auf die Unterarme aufstützen und das rechte Bein nach oben anwinkeln, den Fuß anziehen: Nun langsam das Bein gegen einen Widerstand nach oben schieben, dann wieder lösen und senken
- In der Bauchlage Stirn auf die Hände legen, Beine liegen etwas geöffnet auf dem Boden, Kissen unter das Becken legen, Bauch einziehen und auf einen geraden Rücken achten: Nun das re. Bein etwas anheben (Po anspannen) und senken (mehrmals wiederholen und dann Seitenwchsel); Variation: das re. Bein nach oben anwinkeln und die Fußsohle gegen einen Widerstand hochschieben (Po anspannen)
- Mit Handtuch: In der Rückenlage einen Fuß aufstellen und das Handtuch um das Knie des anderen Beines legen: nun mit Krafteinsatz gegen das Handtuch drücken und die Lendenwirbelsäule gegen den Boden drücken; Variation: Handtuch um beide Knie legen
- Mit elastischem Band: zwei Teilnehmer sitzen sich gegenüber auf dem Stuhl, das elastische Band wird um das Stuhlbein vom Partner

gegenüber gebunden und um den unteren Teil des Unterschenkels: nun das Bein im Kniegelenk beugen und gegen den Widerstand des Bandes arbeiten

Knieextensoren:

- Kniebeugen: Die Teilnehmer stellen sich hinter einen Stuhl/Wandstange/Sprossenwand und halten sich fest. Sie setzen die Beine weit (über hüftbreit) auseinander, die Füße zeigen leicht nach außen. Nun langsam das Gesäß nach hinten unten verschieben, als wolle man sich hinsetzen. Die Knie dürfen dabei nicht nach vorne über die Fußspitzen geschoben werden. Variation: Absenken bis kurz vor einen Stuhl (nicht absetzen!)
- Stufensteigen: mit re. Fuß auf einen kleinen Kasten steigen, dann mit dem li., dann mit dem re. zuerst wieder herunter, dann mit dem li. und umgekehrt.
- mit Handtuch: Im Sitzen auf dem Stuhl beide Füße hüftbreit, Rücken anlehnen, das Handtuch an den Enden fassen: Ein Bein anziehen und das Handtuch um die Fußsohle legen: nun mit Krafteinsatz das Bein strecken, lösen und das Bein wieder beugen; Variation: strecken und beugen gegen ständigen Widerstand
- mit Handtuch: In der Rückenlage Füße hüftbreit aufstellen, ein Kissen unter den Kopf und das Handtuch um eine Fußsohle legen: nun das Bein beugen und einen Widerstand aufbauen (Fuß gebeugt in das Handtuch drücken), den Kopf dabei evtl. anheben; Variation: Fuß nach oben in Richtung Decke drücken
- Mit Handtuch: In der Rückenlage Füße hüftbreit aufstellen, ein Kissen unter den Kopf und das Handtuch über die Unterschenkel (Mitte/unten) legen: nun das Handtuch nach unten ziehen und die Unterschenkel gegen den Widerstand nach oben drücken
- Mit elastischem Band: Teilnehmer sitzt auf dem Stuhl, das elastische Band wird um ein Stuhlbein und um den unteren Teil des Unterschenkels des Beines auf der Seite vom Stuhlbein gebunden: nun das Bein Strecken und gegen den Widerstand des Bandes arbeiten

Hüftbeuger:

- Auf einem Stuhl sitzend, vor den Füßes ein Gegenstand (ca. 20cm hoch, z.B. 0,5l Flasche): nun einen Fuß über den Gegenstand heben und abwechselnd auf beiden Seiten 10mal auf mit der Ferse den Boden berühren; Variation: Übung nicht im Sitzen durchführen sondern im Langsitz auf einer Matte, Hände stützen hinter dem Rücken auf den Unterarmen, der Gegenstand befindet sich ungefähr auf Wadenhöhe

Hüftabduktoren:

- In der Seitenlage, Kopf auf Hand gestützt, nun oberes Bein ca. 50 cm anheben, einen Moment halten und wieder senken; Variation: Steigerung durch elastisches Band als Schlaufe um die Füße
- Mit elastischem Band: In der Seitenlage, Kopf auf Hand gestützt, Beine angewinkelt, Schlaufe um die Füße, nun ein Bein anheben, einen Moment halten und wieder senken

Klinik für Neurologie, Universitätsklinikum Schleswig-Holstein,
Campus Kiel, Schittenhelmstr. 10, D – 24105 Kiel

Universitätsklinikum
Schleswig-Holstein
Campus Kiel

Klinik für Neurologie
Direktor Prof. Dr. med. G. Deuschl
Ansprechpartner: C. Schlenstedt / Prof. Dr. G. Deuschl
Tel: 0431 / 597-8819
Privat: 0431 / 386 70 797
E-Mail: c.schlenstedt@neurologie.uni-kiel.de
Internet: www.uni-kiel.de/neurologie

Informationsblatt zur Studie:

Trainierbarkeit der Gleichgewichtsfähigkeit bei Parkinsonpatienten

Liebe Parkinsonpatienten,

im Herbst 2011 werden ein Gleichgewichtstraining und ein Krafttraining für Parkinsonpatienten angeboten. Durch das Training soll die Sturzgefahr vermindert werden und die Standsicherheit verbessert. Das Training wird im Rahmen der Studie „Trainierbarkeit der Gleichgewichtsfähigkeit bei Parkinsonpatienten" durchgeführt. Hierbei soll untersucht werden, in wieweit sich die Sturzgefahr und die Gleichgewichtsfähigkeit durch Bewegungstherapie verbessern lassen. Dadurch sollen wichtige Erkenntnisse zur Optimierung der Physiotherapie für Parkinsonpatienten gewonnen werden. Eine Teilnahme bietet durch ein gezieltes Übungsprogramm die Chance, den Einschränkungen der Lebensqualität durch Stürze und Angst vor Stürzen entgegenzuwirken.

Trainingszeitraum:	*8 Wochen, zweimal wöchentlich*
Beginn:	*Anfang Oktober*
Ort:	*Physiotherapie der Uniklinik Kiel und Sportinstitut Uni Kiel*
Bedingungen:	*Training in Kleingruppen unter Anleitung, keinerlei sportliche Vorerfahrung notwendig; die Teilnahme ist kostenlos und es kann falls erforderlich ein Fahrtkostenzuschuss gezahlt werden*

Bei Interesse an einer Teilnahme oder weiteren Fragen wenden Sie sich gerne an:

Christian Schlenstedt
Klinik für Neurologie des UK-SH, Campus Kiel
Schittenhelmstraße 10
24105 Kiel
Tel.: 0431 597 - 8550
Tel. Privat: 0431 386 70 797
Mail: c.schlenstedt@neurologie.uni-kiel.de

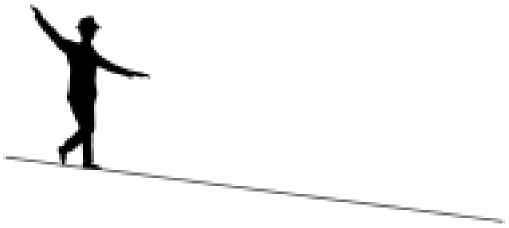

Klinik für Neurologie, Universitätsklinikum Schleswig-Holstein,
Campus Kiel, Schittenhelmstr. 10, D – 24105 Kiel

Universitätsklinikum
Schleswig-Holstein
Campus Kiel

Klinik für Neurologie
Direktor Prof. Dr. med. G. Deuschl
Ansprechpartner: C. Schlenstedt / Prof. Dr. G. Deuschl
Tel: 0431 / 597-8819
Privat: 0431 / 386 70 797
E-Mail: c.schlenstedt@neurologie.uni-kiel.de
Internet: www.uni-kiel.de/neurologie

Information und Aufklärung zur Studie:

Trainierbarkeit der Gleichgewichtsfähigkeit bei Parkinsonpatienten

Liebe Parkinsonpatienten und liebe gesunde Probanden,

hiermit bieten wir Ihnen an, an der Studie „Trainierbarkeit der Gleichgewichtsfähigkeit bei Parkinsonpatienten“ teilzunehmen. Bei dieser Studie wird untersucht, in welchem Maße sich durch Bewegungstherapie die Gleichgewichtsfähigkeit verbessern und das Sturzrisiko verringern lässt. Dafür wird ein sensomotorisches Training (Gleichgewichtstraining) und ein Krafttraining mit Parkinsonpatienten durchgeführt. Hierbei wird verglichen, welche Trainingsform effektiver ist. Außerdem wird auch eine Gruppe gesunder älterer Menschen sensomotorisches Training durchführen. Dadurch ist es möglich zu vergleichen, ob sich Parkinsonpatienten im gleichen Maße wie Gesunde durch sensomotorisches Training verbessern. Durch diese Studie sollen wichtige Erkenntnisse zur Optimierung der Physiotherapie für Parkinsonpatienten gewonnen werden. Eine Teilnahme bietet durch ein gezieltes Übungsprogramm die Chance, den Einschränkungen der Lebensqualität durch Stürze und Angst vor Stürzen entgegenzuwirken.

Das Training wird in Kleingruppen (6-8 Personen) unter Anleitung stattfinden. Die Parkinsonpatienten werden entweder am sensomotorischem Training oder am Krafttraining teilnehmen. Sie werden nach dem Zufallsprinzip der Trainingsform zugeteilt. Das Training wird im Zentrum für Physiotherapie und Physikalische Therapie (Michaelisstraße 1, am Klaus-Groth-Platz in der Klinik für Orthopädie), im Physiotherapieraum der Neurologie (Arnold-Heller-Straße 3) und im Institut für Sport und Sportwissenschaft der Universität Kiel (Olshausenstraße 74) stattfinden. Es wird über einen Zeitraum von 8 Wochen zweimal

wöchentlich trainiert. Eine regelmäßige Teilnahme ist dabei wichtig. Die Übungsintensität richtet sich nach dem individuellen Leistungsniveau der Teilnehmer. Es sind keinerlei sportliche Voraussetzungen für eine Teilnahme notwendig. Das Verletzungsrisiko der Übungen ist äußerst gering. Allerdings kann bei keinem sportlichen Training eine Verletzungsgefahr vollkommen ausgeschlossen werden. Zu Beginn einer Übungsstunde wird ein Aufwärmtraining durchgeführt, um Verletzungen des Bewegungsapparates zu verhindern.

Um die Leistungsverbesserung zu untersuchen, wird jeweils eine Messung vor und nach dem Trainingszeitraum durchgeführt. Außerdem wird vier Wochen nach Beendigung des Trainings eine Wiederholungsmessung durchgeführt. Bei den Messungen bitten wir Sie, kurze Fragebögen auszufüllen. Außerdem führen wir verschiedene Gleichgewichttests, eine Ganganalyse und eine Kraftmessung der Beinkraft durch. Für einen Teil der Tests müssen kleine Marker auf die Haut geklebt werden. Es handelt sich um Kugeln (Durchmesser ca. 2cm) welche mit Klebeband auf die Haut geklebt werden. Dies geschieht schmerzfrei und die Marker werden nach Beendigung der Messung wieder entfernt. Der Gleichgewichtstest und die Ganganalyse werden auf dem Laufband durchgeführt. Um Stürze zu verhindern, tragen die Probanden einen Sicherheitsgurt. Alle durchgeführten Untersuchungen sind nichtinvasiv und schmerzfrei.

Datenschutz:

Um eine Gefährdung für Sie bei der Untersuchung auszuschließen, zur wissenschaftlichen Auswertung und um Kontakt mit Ihnen halten zu können, speichern wir Ihren Namen, Ihr Geburtsdatum, Ihr Geschlecht, Ihre Telefonnummer und Adresse sowie die im Rahmen der Fragebögen und der Aufklärungsgespräche erhobenen medizinischen Daten.

Die Erhebung der Messdaten erfolgt anonymisiert, d.h. in namentlich nicht kenntlicher Form. Zur Auswertung der Messdaten kann es jedoch erforderlich sein, Alter, Geschlecht und für die Auswertung relevante medizinische Daten (z.B. Vorerkrankungen) wieder mit den Messdaten zusammenzuführen. Gemäß der Datenschutzbestimmungen benötigen wir Ihr Einverständnis zur Speicherung und Verwendung dieser Daten im Rahmen unserer Forschungsprojekte.

Fahrtkostenzuschuss:

Für Teilnehmer, die einen längeren Anfahrtsweg haben als 10km, kann ein Fahrtkostenzuschuss von 0,15 € pro Kilometer gezahlt werden.

Parkmöglichkeiten und Parkkosten:

Auf Grund der Umbauarbeiten des Klinikums ist die Parksituation am Universitätsklinikum momentan recht schwierig. Es lassen sich zwei nahe liegende Parkplätze empfehlen (siehe Lageplan):
P1: Vom Schwanenweg kommend nach links auf das Klinikgelände abbiegen in die Michaelisstraße. Auf der rechten Seite (gegenüber der Orthopädie) befinden sich Parkplätze und eine Tiefgarage.

P2: Im Schwanenweg auf der rechten Seite befindet sich das Parkhaus der Kinderklinik.

Die Parkkosten am Klinikum werden erstattet. Es werden hierfür Parkmarken ausgeteilt. Am Institut für Sportwissenschaft ist das Parken kostenlos.

Sollten Sie noch weitere Fragen zur Studie haben, wenden Sie sich gerne an

Christian Schlenstedt
Klinik für Neurologie des UK-SH
Campus Kiel
Schittenhelmstraße 10
24105 Kiel
Tel.: 0431 597-8819
Mail: c.schlenstedt@neurologie.uni-kiel.de

UK SH

UNIVERSITÄTSKLINIKUM
Schleswig-Holstein
→ **Campus Kiel**

6 Allgem. Innere Medizin B1
12 Anästhesiologie B2
25 Augenklinik Hauptgebäude D2
39 Cafeteria C1
18 Chirurgie *Allgemeine C1
5 Exzellenz-Zentrum Endzündungsmedizin A1
24 Frauenklinik (Gynäkologie u. Geburtshilfe) D2
19 Hautklinik (Dermatologie, Venerologie u. Allergologie) B0
27 HNO D1
10 Humangenetik B2
18 Herz- u. Gefäßchirurgie C1
17 Immunologie (Alte Chrirurgie) B2
32 Infektionsmedizin E1
6 Kardiologie B1
9 Kinderklinik (Allgem. Pädiatrie, Kinderkardiologie, Neuropädiatrie) B2
6 Medizin I. Hauptgebäude B1
32 Medizinaluntersuchungsamt u. Krankenhaushygiene E1
14 Molekulare Tumorpathologie C2
26 Mund-, Kiefer- u. Gesichtschirurgie D2
13 MRT (Diagnostische Radiologie) B2
6 Nieren- u. Hochdruckkrankheiten B1
41 Neurozentrum (Neurochirurgie, Neurologie, Neuroradiologie) B1
23 Nuklearmedizin C2
11 Orthopädie B2
14 Pathologie C2
23 Radiologie C2
28 Rechtsmedizin D1
12 Schmerzambulanz Anästhesiologie B2
8 Seelsorge B2
36 Sehschule D2
28 Sexualmedizin D1
43 Stammzell- und Immuntherapie (Med. II. / Dr. Mildred Scheel Haus) A1
23 Strahlentherapie C2
17 Transfusionsmedizin / Blutspende C2
18 Unfallchirurgie C1
18 Urologie C1
31 Verwaltung/Vorstand E1
26 Zahnklinik (Zahnerhaltung u. Paradontologie, Kieferorthopädie u.ä.) D2

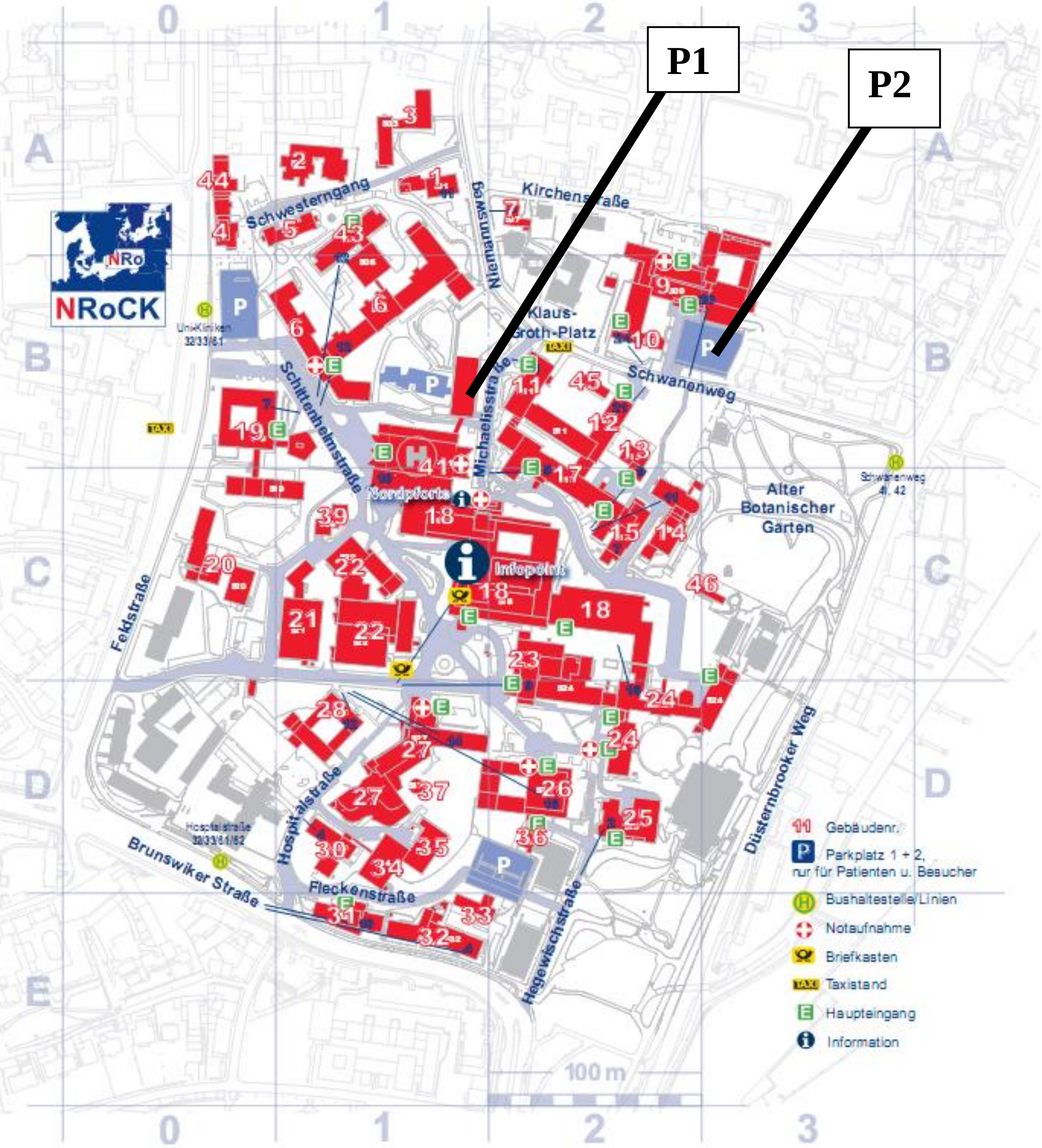

Stand 25.08.2011, Stabsstelle Int. Kommunikation, G. Weinberger, Tel. HL-5033

Klinik für Neurologie, Universitätsklinikum Schleswig-Holstein,
Campus Kiel, Schittenhelmstr. 10, D – 24105 Kiel

Universitätsklinikum
Schleswig-Holstein
Campus Kiel

Klinik für Neurologie
Direktor Prof. Dr. med. G. Deuschl
Ansprechpartner: C.Schlenstedt / Prof. Dr. G. Deuschl
Tel: 0431 / 597-8550
E-Mail: c.schlenstedt@neurologie.uni-kiel.de
Internet: www.uni-kiel.de/neurologie

Einverständniserklärung zur Teilnahme an der Studie:

Trainierbarkeit der Gleichgewichtsfähigkeit bei Parkinsonpatienten

____

Vor- und Nachname der Teilnehmerin / des Teilnehmers

Ich bin ausreichend in mündlicher und schriftlicher Form über die Ziele und Methoden, die möglichen Risiken und den Nutzen der Studie informiert worden. Ich habe die Patienteninformation gelesen und den Inhalt verstanden.
Ich hatte ausreichend Gelegenheit, die Studie mit meinem Arzt zu besprechen und Fragen zu stellen. Alle meine Fragen und Bedenken wurden zu meiner Zufriedenheit beantwortet.
Ich weiß, dass meine Studienteilnahme freiwillig ist und dass ich jederzeit ohne Angabe von Gründen meine Zusage zur Teilnahme zurückziehen kann und mir daraus für meine weitere Behandlung keine Nachteile entstehen.
Ich bin damit einverstanden, dass im Rahmen der Studie meine Daten und Krankheitsdaten aufgezeichnet und anonymisiert (d.h. ohne Namensnennung) zur Auswertung der Ergebnisse verwendet werden. Alle im Rahmen der Studie erhobenen Daten werden strikt vertraulich gemäß dem Datenschutz behandelt.
Einer wissenschaftlichen Auswertung der anonymisierten Daten und einer Veröffentlichung der Ergebnisse stimme ich zu.
Ich gebe hiermit meine freiwillige Zustimmung zur Teilnahme an dieser Studie. Eine Kopie dieser Einwilligung und eine Kopie der Patienteninformationsschrift habe ich erhalten.

____ __________

Name der aufklärenden Person (in Druckbuchstaben) und Unterschrift Datum

_________________________________ __________

Unterschrift der Patientin/ des Patienten Datum

Studienoutline

**Titel**	**Trainierbarkeit der posturalen Kontrolle bei Parkinsonpatienten**
**Studienleiter**	Prof. Dr. G. Deuschl / C. Schlenstedt / Dr. J. Raethjen<br>Klinik für Neurologie<br>UK-SH, Campus Kiel<br>Schittenhelmstrasse 10<br>24105 Kiel
**Hauptzielkriterium**	Vergleich der Effekte von sensomotorischem Training mit Krafttraining hinsichtlich der Verbesserung der posturalen Kontrolle bei Parkinsonpatienten; Vergleich der motorischen Reserve von Parkinsonpatienten und Gesunden hinsichtlich sensomotorischem Training.
**Design**	Parkinsonpatienten: zweiarmig, randomisiert + Gesunde
**Patientenzahl**	60 (40 Parkinsonpatienten + 20 Gesunde) : 20 – 20 – 20
**Patientenselektion**	Parkinsonpatienten: diagnostizierter Morbus Parkinson, posturale Instabilität
**Randomisierung**	Extern
**Behandlung**	<u>Parkinsongruppe 1:</u> 8 Wochen, zweimal wöchentlich: sensomotorisches Training (Gleichgewichtsübungen)<br><u>Parkinsongruppe 2:</u> 8 Wochen, zweimal wöchentlich: Krafttraining (Kraftigungsübungen mit Kleingeräten)<br><u>Gesunde:</u> 8 Wochen, zweimal wöchentlich: sensomotorisches Training (Gleichgewichtsübungen)
**Erhebungszeitpunkte**	**1** Baseline vor dem Trainingszeitraum<br>**2** unmittelbar nach der 8-wöchigen Trainingsphase<br>**3** follow-up: 4 Wochen nach Beendigung des Trainings
**Studienendpunkte**	- Berg Balance Scale<br>- Sturzkalender<br>- Beinkraftmessung (Explosivkraft + Maximalkraft)<br>- Dynamische Posturographie<br>- Ganganalyse<br>- UPDRS Teil 2 und 3<br>- Freezing Score<br>- Freezing of Gait Questionnaire (FOGQ)<br>- Analyse der Ganginitiierung<br>- PDQ-39
